# Cuproptosis-Related Ferroptosis genes for Predicting Prognosis in kidney renal clear cell carcinoma

**DOI:** 10.1186/s40001-023-01137-z

**Published:** 2023-05-15

**Authors:** Gang Luo, Lini Wang, Ziyu Zheng, Baobao Gao, Chong Lei

**Affiliations:** Department of Anesthesiology and Perioperative Medicine, Xijing Hospital, The Fourth Military Medical University, Xi’an, 710032 China

**Keywords:** Signature, Clear Cell Renal Cell Carcinoma, Cuproptosis, Ferroptosis

## Abstract

Kidney renal clear cell carcinoma (KIRC) is a main subtype of kidney cancers. Cuproptosis and ferroptosis are correlated with immune infiltration and prognosis in tumors. However, the role of Cuproptosis-related Ferroptosis genes (CRFGs) in KIRC has rarely been fully understood. Therefore, we constructed a prognostic signature based on different expression of CRFGs in KIRC. All raw data of this study were extracted from public TCGA datasets. Cuproptosis and Ferroptosis genes were collected from the previous research. Finally, a total of 36 significantly different CRFGs were identified from TCGA–KIRC cohort. Six-gene signature (*TRIB3*, *SLC2A3*, *PML*, *CD44*, *CDKN2A* and *MIOX*) was identified by LASSO Cox regression based on the significantly different CRFGs. The CRFGs signature was correlated with worse overall survival and the AUC was 0.750. Functional enrichment indicated that CRFGs were mainly enriched in metabolism, drug resistance, tumor immunity pathways. Besides, the IC50 and immune checkpoint differentially expressed between different groups. The proposed 6-CRFGs signature is a promising biomarker to predict clinical outcomes and therapeutic responses for KIRC patient.

**Supplementary Information:**

The online version contains supplementary material available at 10.1186/s40001-023-01137-z.

## Introduction

Kidney cancer, accounting for approximately 4.2% of all malignancies, is a disease affecting human health and life [1]. Kidney renal clear cell carcinoma (KIRC) represents approximately 85% of Renal cell carcinoma (RCC) [2, 3]. Targeted treatment, chemotherapy, and immunotherapy are preferred therapy for advanced clear cell carcinoma [4]. However, the therapeutic outcomes are proved to be of unsatisfactory due to the lack of individual variance, reliable prognostic biomarkers and medication resistance [5]. Therefore, it is crucial to seek accurate predictive biomarkers to improve prognosisis of KIRC patients.

Studies demonstrated that various cell death modalities were closely involved in cancer eradication. Cuproptosis is a newly discovered mechanism of copper-induced cell death [6–9]. Evidence suggests that copper-induced cell death is vital to cancer progression, such as lung [10], thyroid [11], gallbladder [12], breast [13], and prostate [14]. Meanwhile, Ferroptosis is another type of iron-reliant cell death by reactive oxygen species (ROS) accumulation, but differs from autophagy pyroptosis, necrosis and apoptosis [15, 16]. Inhibition ferroptosis may be a promising strategy for cancer therapies, such as ovarian and lung cancer [17, 18]. Recently, it has been suggested excessive copper increased iron toxicity and the development of oxidative stress [19]. In contrary, recent investigations reported that copper affected iron metabolism in neurodegenerative diseases, immunological diseases, and cancer [20–22]. In addition, some studies have shown that CuO can promote apoptosis and cytotoxicity modified by reactive oxygen species (ROS) [23, 24]. The abovementioned evidence suggests that copper–iron interactions participants in various physiology and pathophysiology process including cancer progression. However, few studies have focused on copper–iron interactions in terms of cuproptosis–ferroptosis interaction in KIRC.

Here, we established a Cuproptosis-related Ferroptosis genes (CRFGs) signature. We also characterized the immune and molecular profile of CRFGs signature. The signature was effective for predicting KIRC prognosis.

## Methods

### Data collection and diferentially expressed gene analysis

**Fig. 1:**
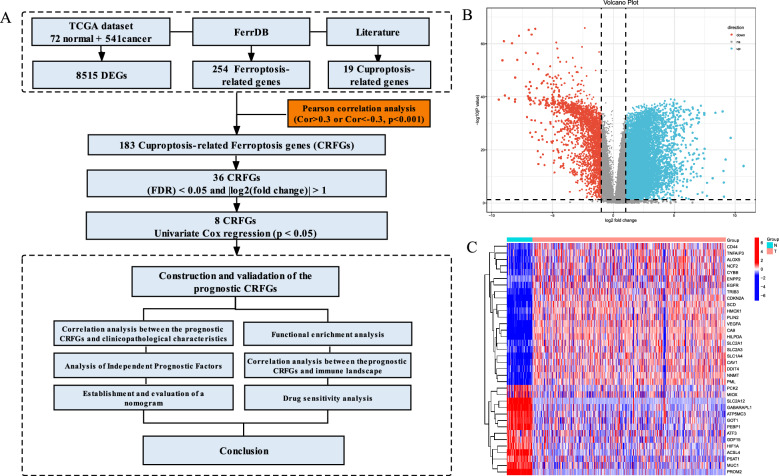
**A** The flowchart of this study; **B** Volcano plot showed differentially expressed genes between normal and cancer tissues of KIRC in TCGA datasets; **C** Heatmap showed differentially expressed CRFGs in TCGA datasets

All row data of this study were extracted from public database of The Cancer Genome Atlas (TCGA) repository (72 normal samples and 541 tumor samples). All data are publicly available and open access. Therefore, the institutional review board approval was waived. The flow diagram for this study is displayed in Fig. 1A. A detailed clinical characteristics is given in Table 1. Ferroptosis and cuproptosis genes collected from the previous studies and FerrDb database [25, 26]. Finally, all genes are listed in Additional file 1: Table S1, respectively. Differentially expressed genes (DEGs) between nontumor and KIRC tissues were identified. The Pearson correlation coefficient was relationship between the cuproptosis and ferroptosis genes was calculated with *p* < 0.001 and correlation coefficient |r^2^|> 0.3.

**Table 1:** Gene list and coefficient

Gene symbol	Coefficient
TRIB3	0.1430
SLC2A3	0.0296
PML	0.3216
CD44	0.1135
CDKN2A	0.2139
MIOX	-0.0496

### Construction the cuproptosis-related ferroptosis gene signature

We developed a CRFGs signature by least absolute shrinkage and selection operator (LASSO) Cox regression. The prognostic gene signature was built by parameter λ with tenfold cross-validation. Patients were divided into KIRC-high and KIRC-low group. Prediction efficacy was evaluated with time-dependent ROC. Furthermore, a nomogram was generated to visually predict specific outcomes. Besides, the calibration and discrimination of the nomogram was assessed respectively. The GSE29609 data sets were used as external validation for the CRFGs score prediction model.

### Molecular mechanisms

The protein–protein interaction (PPI) network, KEGG (Kyoto Encyclopedia of Genes and Genomes) pathway and GO (Gene ontology) were used to predict molecular mechanisms of CRFGs.

### Drug sensitivity analysis and immune cell infiltration

We assessed tumor-infiltrating immune cell by EPIC algorithms, CIBERSORT-ABS, MCP-counter, XCELL, QUANTISEQ, CIBERSORT, and TIMER. We also compared immune checkpoints and drug sensitivity from KIRC-high and KIRC-low group.

### Statistical analysis

All analyses were done in R software (R 4.1.3). Continuous variables were compared by T-test. Survival analysis was performed by log-rank test. Statistical significance was set *p* < 0.05.

## Results

### Identification of CRFGs

The baseline clinical of KIRC patients in TCGA dataset is listed in Table 1. In total, 8515 DEGs were identified between KIRC nontumor and tumor tissues (Fig. 1B). By analyzing the correlation coefficient of cuproptosis-related ferroptosis genes, we obtained 183 cuproptosis-related ferroptosis genes (*p* < 0.001 and |Cor|> 0.3). Subsequently, we screened 36 cuproptosis-related ferroptosis differential DEGs (Fig. 1C). As expected, cuproptosis-related ferroptosis genes were different from KIRC normal and tumor tissues.

### Establishment of CRFGs signature

**Fig. 2:**
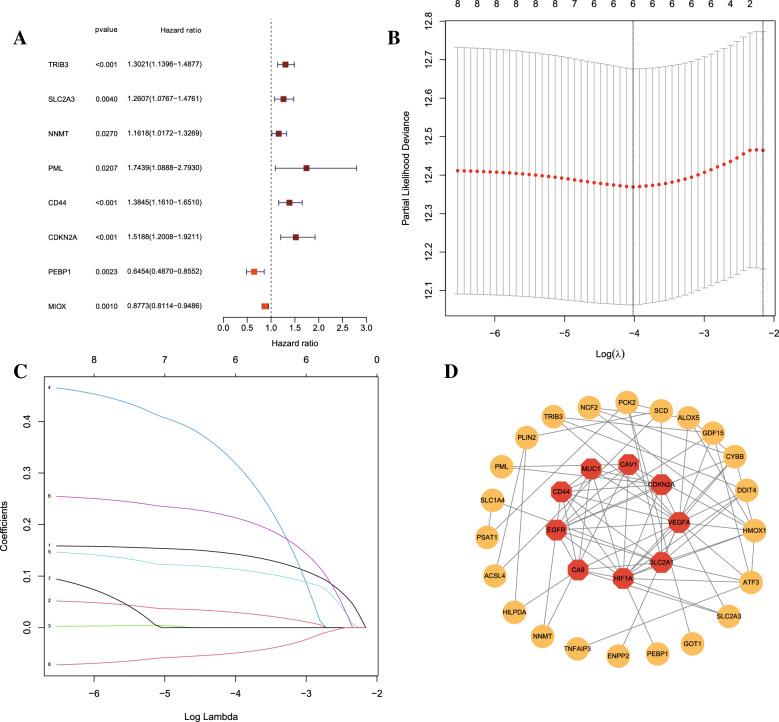
**A** Identification of prognostic CRFGs by univariate Cox regression analysis; **B**, **C** The least absolute shrinkage and selection operator (LASSO) regression was performed with the minimum criteria; **D** Protein–protein interaction network of differentially expressed CRFGs

**Fig. 3:**
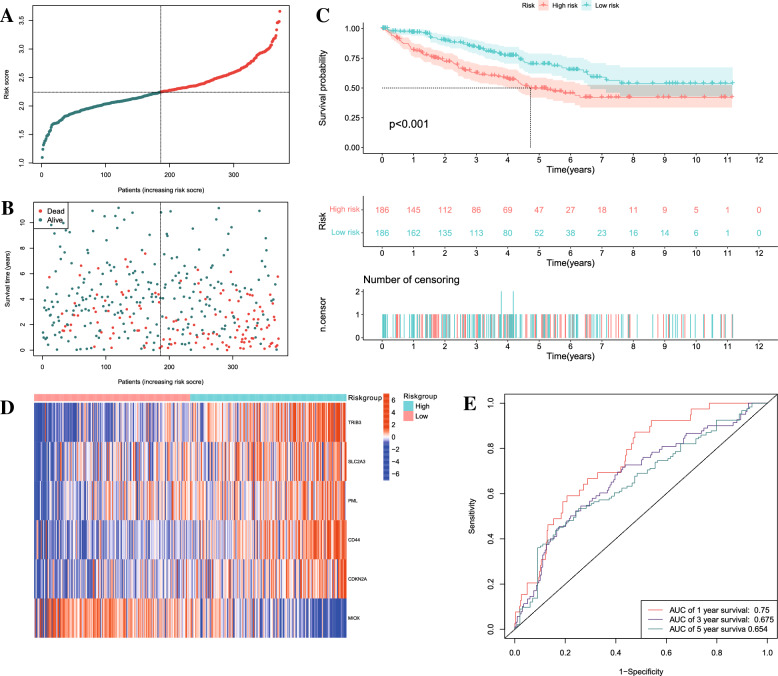
**A**, **B** Distribution of survival status based on the median risk score in TCGA set. **C** Kaplan–Meier survival analysis of KIRC patients between high-risk groups and low-risk groups; **D** Heatmap showed the differences of 6 CRFGs between high-risk and low-risk in KIRC patients; **E** The receiver operating characteristic (ROC) curve analyses of the prognostic CRFGs in predicting 1-, 3-, and 5-year overall survival (OS)

To explore whether CRFGs correlated to KIRC patient prognosis, the aforementioned 36 identified genes were analyzed by univariate Cox regression (Fig. 2A). It was demonstrated that eight cuproptosis-related ferroptosis genes with *p* < 0.05 were correlated to the KIRC survival. Later, a six-gene prognostic signature was generated by LASSO Cox regression using the expression value of the above mentioned 8 prognostic genes (Fig. 2B-C). The selected six genes were *TRIB3, SLC2A3, PML, CD44, CDKN2A* and *MIOX*. The risk score was calculated by relevant coefficient of 6 prognostic signature genes (Table 2). Risk score = (0.1430 × TRIB3 profile) + (0.0296 × *SLC2A3* profile) + (0.3216 × *PML* profile) + (0.1135 × *CD44* profile) + (0.2139 × *CDKN2A* profile) + (-0.0496 × *MIOX* profile). The result of PPI network showed *CD44, EGFR, CA9, HIF1A, SLC2A, VEGFA, CDKN2A, CAV1* and *MUC1* were hub genes(Fig. 2D). Then, patients were categorized into KIRC-low and KIRC-high groups. KIRC-high group exhibited poor survival outcome than KIRC-low group (Fig. 3A–C). The expression of signature varies across low- and high-score group (Fig. 3D). The ROC curve of 1-, 3,- and 5-year OS was 0.75, 0.675 and 0.654 (Fig. 3E). Similarly, the signature showed powerful forecasting capability for KIRC survival outcomes in validation cohorts (Additional file 2: Figure S1).

**Table 2:** The clinical characteristics of patients in the TCGA dataset

Variable	Number of samples
Gender	
Male	346
Female	191
Age at diagnosis	
≤ 65	352
> 65	185
Grade	
G1	14
G2	230
G3	207
G4	78
NA	8
Stage	
I	269
II	57
III	125
IV	83
NA	3
T	
T1	275
T2	69
T3	182
T4	11
M	
M0	426
M1	79
NA	32
N	
N0	240
N1	17
NA	280

### Analysis of independent prognostic factors

**Fig. 4:**
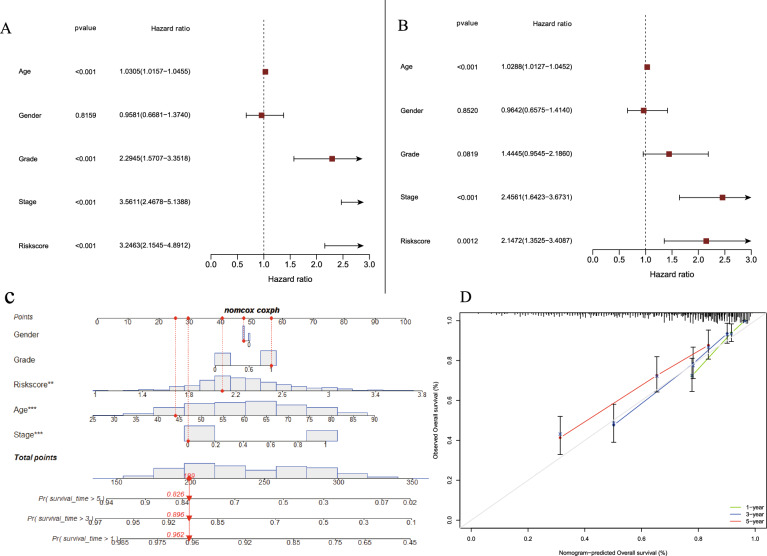
**A** The correlations between the risk score and clinicopathological factors by univariate Cox regression analysis; **B** The correlations between the risk score and clinicopathological factors by multivariate Cox regression analysis; **C**, **D** the nomogram and calibration curve of the CRFGs-score model, respectively

Univariate regression found that stage, age, risk score, and Grade were related to the survival of KIRC patients (*p* < 0.001) (Fig. 4A). Further, multivariate regression indicated that the age, stage and risk score were also correlated to KIRC survival (*p* < 0.05) (Fig. 4B). These results revealed that CRFGs signature could serve as prognostic marker of KIRC.

### Correlation between the signature and clinical characteristics

Among the signature, five genes (*TRIB3*, *SLC2A3*, *PML*, *CD44* and *CDKN2A*) unregulated in high-risk group of KIRC. The *MIOX* gene down-regulated in high-risk group and may be a protective genes (Additional file 3: Figure S2 A). The analysis of clinical data showed that N stage, gender, M stage, T stage and grade were different from these two groups (*P* < 0.05) (Additional file 3: Figure S2 B-G). However, CRFGs-based signature had poor predicted outcomes in M stage (M0, M1), T stage (T1-T4), stage (stageI-IV) and Grade (G1-G2) (*P* > 0.05) (Additional file 4: Figure S3. A-E). Moreover, a CRFGs risk score-based nomogram was developed as graphical calculators for predicting the prognosis in KIRC patients. The nomogram performed good calibration and accuracy (Fig. 4C-D). Therefore, the nomogram may be used in the evaluation of KIRC patients.

### Functional enrichment

**Fig. 5:**
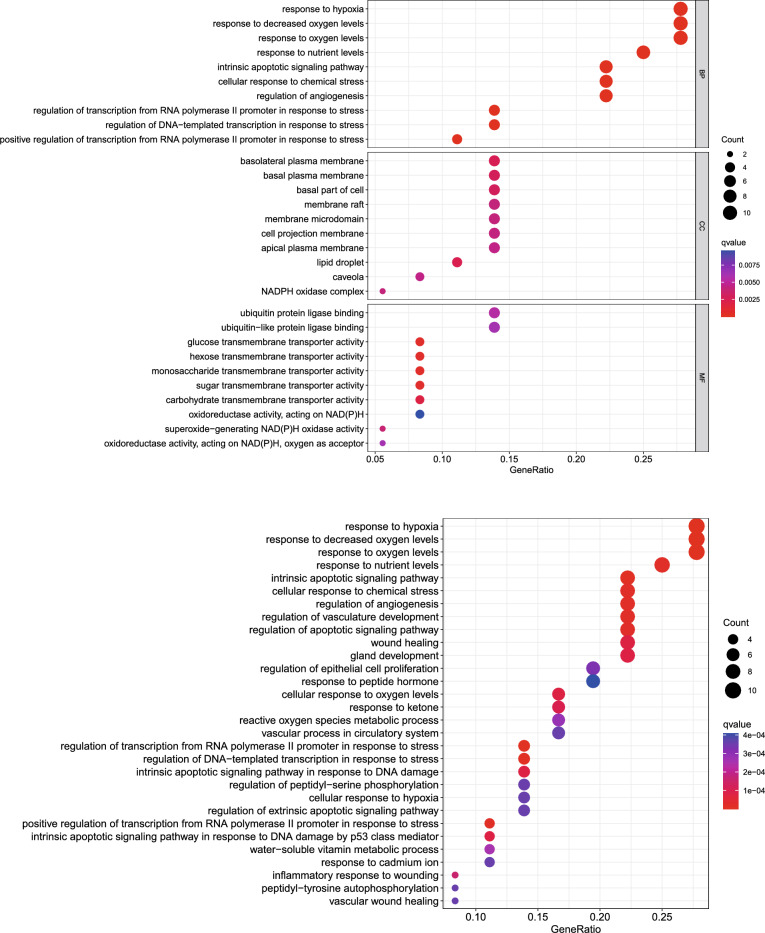
(**A**, **B**) Go analysis and KEGG analysis respectively

KEGG and GO analysis showed a significant enrichment in intrinsic apoptotic, response to oxygen levels, DNA damage, apoptotic, angiogenesis, nutrient levels and chemical stress (Fig. 5B). In summary, this signature is related to biological metabolism, drug resistance, tumor immunity and metastasis in KIRC.

### Immune infiltration and drug sensitivity analysis

**Fig. 6:**
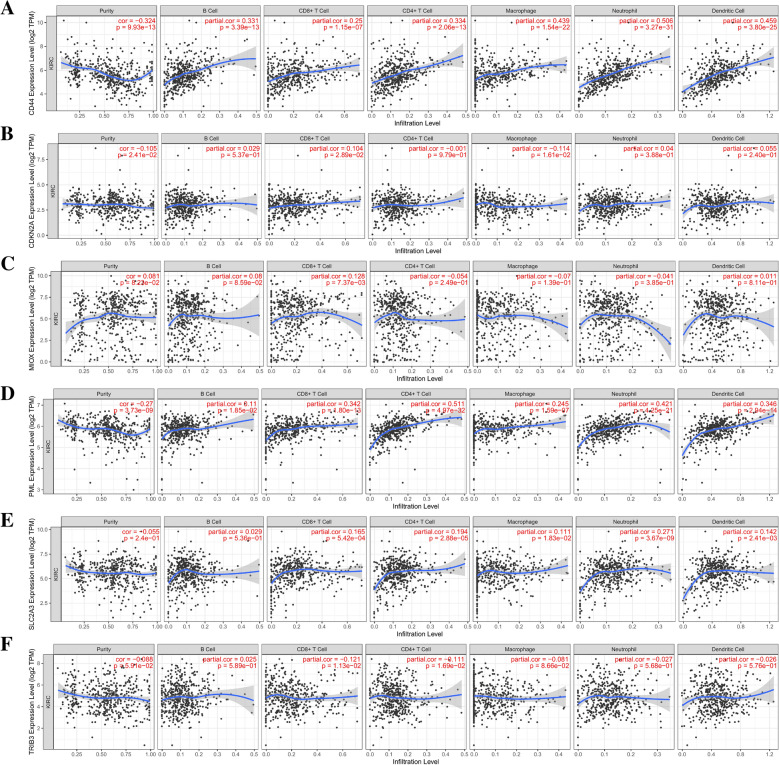
Correlation between **A** CD44, **B** CDKN2A, **C** MIOX, **D** PML, **E** SLC2A3 and **F** TRIB3 expression and immune cell in KIRC in the TIMER database

The immune infiltration of KIRC-high and KIRC-low is displayed in Additional file 5: Figure S4. The immune checkpoint of ICOS, BTLA, CTLA-4, CD27, CD28, CD40, LAG3, PVR, SIRPA, TIGIT, HLA-DPA1 and TNFRSF9, was upregulated in KIRC-high group (Additioanl file 6: Figure S5). Immune infiltration showed the level of *CD44* and *PML* correlated positively to dendritic Cells, CD4-CD8 + T cells and B cells. *SLC2A3*, *PML* and *CD44* were positively related to macrophages and neutrophils; The *CDKN2A* expression level correlated negatively to macrophages. *TRIB3* was negatively associated with CD4-CD8 + T cells (Fig. 6A-F). The drug sensitivity analysis found that RDEA119, Bexarotene, Bicalutamide, Bortezomib, Cyclopamine, Embelin, Midostaurin, Dasatinib, Thapsigargin, Sorafenib, Salubrinal, and Obatoclax Mesylate were higher in low- than high-risk group (Additional file 7: Figure S6).

## Discussion

In this study, we established a prognostic model that incorperate six-gene signature of CRFGs and clinical features of KIRC patients to predict survival. The developed model had moderate discrimination and good calibration abilities in predicting survival. A robust association of CRFGs signature and KIRC prognosis patients was verified by functional enrichment, immune infiltration, immune checkpoint, tumor micro-environment and drug sensitivity analyses.

The KIRC patients’ prognosis is related to clinical index, genes, proteins and so on. Cuproptosis and Ferroptosis are new types of cell death mechanisms [26, 27]. All the cuproptosis and ferroptosis-related genes are considered to be promising anti-tumor targets [28–30]. Emerging research suggested that cuproptosis and ferroptosis were key factors of KIRC development. To date, based on cuproptosis and ferroptosis, multiple biomarkers with fair prediction performance for predicting prognostic mRNA or lncRNA value in KIRC have been established [26, 31–33]. However, the association of CRFGs and prognosis of KIRC needs to be investigated, as developing a CRFGs signature for predicting KIRC prognosis and optimizing therapeutic methods is speculated to be helpful.

The prognostic signature was screened in this study (T*RIB3, SLC2A3, PML, CD44, CDKN2A* and *MIOX*). *TRIB3* could inhibit mitosis in germ cell [34]. *TRIB3 can* promote cell proliferation in Renal Cell Carcinoma Cells by MAPK Signaling Pathway [35]. Moreover, *TRIB3* induces immune evasion and reduces CD8^+^ T cell infiltration in colorectal cancer [36]. In breast cancer, *TRIB3* supports cell stemness by regulation of *SOX2* transcription [36]. *SLC2A3, a membrane proteins,* could inhibit tumor growth by up-regulating miR-184 [37]. In gastric cancer, *SLC2A3* promotes infiltrating macrophages [38]. *PML* is a tumor suppressor response to environmental stimuli and crucial to antiviral defense activities [39]. *CD44*, known as P-glycoprotein 1, has been associated with tumor metastasis and invasion [40, 41]. Our study investigated the prognostic value of six CRFGs in KIRC. However, more intensive researches are warranted to explore potential regulatory effects for KIRC.

The functional enrichment revealed that the CRFGs related to many immune-related biological processes and pathways. Renal cell carcinomas (RCCs) highly resistant against chemotherapies, which may be due to impaired intrinsic or extrinsic apoptotic pathways [42, 43]. p53, a key regulator response to DNA damage, associated with poor patient prognosis and aggressiveness of tumor [44, 45]. Interestingly, p53 is inactivated in KIRC and considered as inducer for DNA damage response [46].

Cuproptosis and ferroptosis are also considered a form of immunogenic cell death [47]. Tumor microenvironment (TME) is a novel factor for cancer treatment [48]. Our results showed that Treg cells and tumor-associated macrophages (TAMs) were abundant in KIRC-high group than KIRC-low group. The CD8^+^T cells are vital to tumor progression and have antitumor effect.[49]. In KIRC, CD8^+^T cells was a favorable prognostic factor [50]. Our results are consistent with this conclusion. Moreover, We witnessed the expression of immune checkpoint, including CTLA-4, BTLA, CD27, CD28, CD40, ICOS, LAG3, PVR, SIRPA, TIGIT and TNFRSF9, was higher in high risk group might be owing to the immunosuppressive microenvironment. Immune checkpoint inhibitors activate the immune cell to kill cancer cells [51]. Ipilimumab, an anti CTLA-4 drugs, was used in KIRC patients [52]. All the results revealed that the signature can be further developed to evaluate the efcacy of KIRC patients.

Predicting the drug sensitivity promoted in improving drug selectivity and increasing the success rate of therapy [53]. Surprisingly, high-risk group patients were more susceptible to RDEA119, Bexarotene, Bicalutamide, Bortezomib, Cyclopamine, Embelin, Midostaurin, Dasatinib, Thapsigargin, Sorafenib, Obatoclax. Mesylate, and Salubrinal. In early study, cyclopamine was safe and well tolerated by the mice [54]. In glioblastoma, Cyclopamine acts to suppress carcinogenesis [55]. This may provide novel therapeutic strategies in KIRC patients.

Cell death has been implicated to cancer development [56]. Cuproptosis and ferroptosisis are two distinct regulated cell death mechanisms. Such unusual mechanisms may lead to a new therapeutic opportunity for treating cancer. In our study, the predictive value of CRFGs was comparable in KIRC-high and KIRC-low group. Nonetheless, it also has some limitations. First, whether this CRFGs signature can modulate KIRC process remains unknown. Their function needs further exploration. Second, the utility of prognostic model in this study requires further validation by a large sample size based on prospective studies in future research. Last but not least, cuproptosis and ferroptosis are new fields of cancer research. All genes were used in our study may be incomplete due to more and more these genes will be discovered.

In conclusion, we established a novel CRFGs signature that can predict KIRC prognosis and further studies are needed for validation of signature.

## Supplementary Information

**Additional file 1: Table S1.** The gene list of 19 cuproptosis-related genes and 254 ferroptosis-related genes.

**Additional file 2: Figure S1.**Distribution of survival status based on the median risk score in validation set;The receiver operating characteristiccurve analyses of the prognostic CRFGs in predicting 1-, 3-, and 5-year overall survival;Kaplan–Meier survival analysis of KIRC patients between high-risk groups and low-risk groups.

**Additional file 3: Figure S2.** Correlation between signature and clinical characteristics.

**Additional file 4: Figure S3.** Kaplan–Meier curves of OS diferences stratifed by gender, age, grade, N stage, T stage, or M stage between the high-risk groups and low-risk groups.

**Additional file 5: Figure S4.** Immune cells infiltration between high-risk groups and low-risk groups.

**Additional file 6: Figure S5.** The relationship between prognostic signature and immune checkpoints.

**Additional file 7: Figure S6.** Drug sensitivity analysis.

## Data Availability

The data during the current study are available from TCGA and GEO database.

## Abbreviations

RCC: Renal cell carcinoma
CRFGs: Cuproptosis-related Ferroptosis genes
ccRCC: Clear cell renal cell carcinoma
LASSO: least absolute shrinkage and selection operator
KIRC: Renal clear cell carcinoma
TCGA: Cancer genome atlas
TCA: Tricarboxylic acid
ROS: Reactive oxygen species
DEGs: Diferentially expressed genes
GO: Gene ontology
FDR: False discovery rate
BP: Biological processes
CC: Cellular components
MF: Molecular function
PPI: Protein–protein interaction
KEGG: Kyoto Encyclopedia of Genes and Genomes
ROC: Receiver operating characteristic
C-index: Concordance index
TIMER: Tumor immune estimation resource
GDSC: Genomics of Drug Sensitivity in Cancer
CI: Confidence interval
HR: Hazard ratio
TME: Tumor microenvironment
