# Supplementary material for: Cuproptosis-Related Ferroptosis genes for Predicting Prognosis in kidney renal clear cell carcinoma
Source: Eur J Med Res. 2023 May 15;28:176. doi: 10.1186/s40001-023-01137-z (PMC10184413; doi:10.1186/s40001-023-01137-z)
Supplement: Supplementary file 1 — Additional file 1: Table S1. The gene list of 19 cuproptosis-related genes and 254 ferroptosis-related genes. [file 40001_2023_1137_MOESM1_ESM.docx]

**Additional file 1: Table S1.** The gene list of 19 cuproptosis-related genes and 254 ferroptosis-related genes.

| gene symbol | Type | gene symbol | Type | Gene symbol | Type | gene symbol | Type |
| --- | --- | --- | --- | --- | --- | --- | --- |
| NFE2L2 | cuproptosis | BLOC1S5-TXNDC5 | ferroptosis | CISD1 | ferroptosis | GLS2 | ferroptosis |
| NLRP3 | cuproptosis | EIF2S1 | ferroptosis | FANCD2 | ferroptosis | GOT1 | ferroptosis |
| ATP7B | cuproptosis | IL6 | ferroptosis | FTMT | ferroptosis | CARS1 | ferroptosis |
| ATP7A | cuproptosis | CXCL2 | ferroptosis | HSPA5 | ferroptosis | KEAP1 | ferroptosis |
| SLC31A1 | cuproptosis | RELA | ferroptosis | TP53 | ferroptosis | ATG5 | ferroptosis |
| FDX1 | cuproptosis | HSD17B11 | ferroptosis | HELLS | ferroptosis | ATG7 | ferroptosis |
| LIAS | cuproptosis | AGPAT3 | ferroptosis | SCD | ferroptosis | NCOA4 | ferroptosis |
| LIPT1 | cuproptosis | SETD1B | ferroptosis | FADS2 | ferroptosis | ALOX12B | ferroptosis |
| LIPT2 | cuproptosis | HMOX1 | ferroptosis | SRC | ferroptosis | ALOX15B | ferroptosis |
| DLD | cuproptosis | TF | ferroptosis | STAT3 | ferroptosis | ALOXE3 | ferroptosis |
| DLAT | cuproptosis | FTL | ferroptosis | PML | ferroptosis | PHKG2 | ferroptosis |
| PDHA1 | cuproptosis | RPL8 | ferroptosis | MTOR | ferroptosis | ACO1 | ferroptosis |
| PDHB | cuproptosis | ATP5MC3 | ferroptosis | NFS1 | ferroptosis | ULK1 | ferroptosis |
| MTF1 | cuproptosis | TFRC | ferroptosis | TP63 | ferroptosis | ATG3 | ferroptosis |
| GLS | cuproptosis | MAFG | ferroptosis | CDKN1A | ferroptosis | ATG4D | ferroptosis |
| CDKN2A | cuproptosis | IL33 | ferroptosis | MIR137 | ferroptosis | BECN1 | ferroptosis |
| DBT | cuproptosis | FTH1 | ferroptosis | ENPP2 | ferroptosis | MAP1LC3A | ferroptosis |
| GCSH | cuproptosis | SLC40A1 | ferroptosis | VDAC2 | ferroptosis | GABARAPL2 | ferroptosis |
| DLST | cuproptosis | GPX4 | ferroptosis | FH | ferroptosis | GABARAPL1 | ferroptosis |
| PTGS2 | ferroptosis | HAMP | ferroptosis | CISD2 | ferroptosis | ATG16L1 | ferroptosis |
| DUSP1 | ferroptosis | HSPB1 | ferroptosis | MIR9-1 | ferroptosis | WIPI1 | ferroptosis |
| NOS2 | ferroptosis | NFE2L2 | ferroptosis | MIR9-2 | ferroptosis | WIPI2 | ferroptosis |
| NCF2 | ferroptosis | STEAP3 | ferroptosis | MIR9-3 | ferroptosis | SNX4 | ferroptosis |
| MT3 | ferroptosis | DRD5 | ferroptosis | ISCU | ferroptosis | ATG13 | ferroptosis |
| UBC | ferroptosis | DRD4 | ferroptosis | ACSL3 | ferroptosis | ULK2 | ferroptosis |
| ALB | ferroptosis | MAP3K5 | ferroptosis | OTUB1 | ferroptosis | SAT1 | ferroptosis |
| TXNRD1 | ferroptosis | MAPK14 | ferroptosis | CD44 | ferroptosis | EGFR | ferroptosis |
| SRXN1 | ferroptosis | SLC2A1 | ferroptosis | LINC00336 | ferroptosis | MAPK3 | ferroptosis |
| GPX2 | ferroptosis | SLC2A3 | ferroptosis | BRD4 | ferroptosis | MAPK1 | ferroptosis |
| BNIP3 | ferroptosis | SLC2A6 | ferroptosis | PRDX6 | ferroptosis | BID | ferroptosis |
| OXSR1 | ferroptosis | SLC2A8 | ferroptosis | MIR17 | ferroptosis | ZEB1 | ferroptosis |
| SELENOS | ferroptosis | SLC2A12 | ferroptosis | NF2 | ferroptosis | DPP4 | ferroptosis |
| ANGPTL7 | ferroptosis | SLC2A14 | ferroptosis | ARNTL | ferroptosis | CDKN2A | ferroptosis |
| CHAC1 | ferroptosis | EIF2AK4 | ferroptosis | HIF1A | ferroptosis | PEBP1 | ferroptosis |
| SLC7A11 | ferroptosis | ALOX5 | ferroptosis | JUN | ferroptosis | SOCS1 | ferroptosis |
| DDIT4 | ferroptosis | ALOX12 | ferroptosis | CA9 | ferroptosis | CDO1 | ferroptosis |
| ASNS | ferroptosis | ALOX15 | ferroptosis | TMBIM4 | ferroptosis | MYB | ferroptosis |
| TSC22D3 | ferroptosis | ACSF2 | ferroptosis | PLIN2 | ferroptosis | MAPK8 | ferroptosis |
| DDIT3 | ferroptosis | IREB2 | ferroptosis | MIR212 | ferroptosis | MAPK9 | ferroptosis |
| JDP2 | ferroptosis | HMGB1 | ferroptosis | AIFM2 | ferroptosis | LINC00472 | ferroptosis |
| SESN2 | ferroptosis | ELAVL1 | ferroptosis | LAMP2 | ferroptosis | PRKAA2 | ferroptosis |
| SLC1A4 | ferroptosis | TFAP2C | ferroptosis | ZFP36 | ferroptosis | PRKAA1 | ferroptosis |
| PCK2 | ferroptosis | SP1 | ferroptosis | PROM2 | ferroptosis | BAP1 | ferroptosis |
| TXNIP | ferroptosis | HBA1 | ferroptosis | CHMP5 | ferroptosis | ABCC1 | ferroptosis |
| VLDLR | ferroptosis | NNMT | ferroptosis | CHMP6 | ferroptosis | MIR6852 | ferroptosis |
| GPT2 | ferroptosis | PLIN4 | ferroptosis | CAV1 | ferroptosis | ACVR1B | ferroptosis |
| PSAT1 | ferroptosis | HIC1 | ferroptosis | GCH1 | ferroptosis | TGFBR1 | ferroptosis |
| LURAP1L | ferroptosis | STMN1 | ferroptosis | CS | ferroptosis | EPAS1 | ferroptosis |
| SLC7A5 | ferroptosis | RRM2 | ferroptosis | EMC2 | ferroptosis | HILPDA | ferroptosis |
| HERPUD1 | ferroptosis | CAPG | ferroptosis | NOX1 | ferroptosis | IFNG | ferroptosis |
| XBP1 | ferroptosis | HNF4A | ferroptosis | CYBB | ferroptosis | ANO6 | ferroptosis |
| ATF3 | ferroptosis | NGB | ferroptosis | NOX3 | ferroptosis | LPIN1 | ferroptosis |
| SLC3A2 | ferroptosis | YWHAE | ferroptosis | NOX4 | ferroptosis | TNFAIP3 | ferroptosis |
| CBS | ferroptosis | GABPB1 | ferroptosis | NOX5 | ferroptosis | TLR4 | ferroptosis |
| ATF4 | ferroptosis | AURKA | ferroptosis | DUOX1 | ferroptosis | ATM | ferroptosis |
| ZNF419 | ferroptosis | MIR4715 | ferroptosis | DUOX2 | ferroptosis | YY1AP1 | ferroptosis |
| KLHL24 | ferroptosis | RIPK1 | ferroptosis | G6PD | ferroptosis | EGLN2 | ferroptosis |
| TRIB3 | ferroptosis | PRDX1 | ferroptosis | PGD | ferroptosis | MIOX | ferroptosis |
| ZFP69B | ferroptosis | MIR30B | ferroptosis | PIK3CA | ferroptosis | TAZ | ferroptosis |
| ATP6V1G2 | ferroptosis | AKR1C1 | ferroptosis | FLT3 | ferroptosis | MTDH | ferroptosis |
| VEGFA | ferroptosis | AKR1C2 | ferroptosis | SCP2 | ferroptosis | IDH1 | ferroptosis |
| GDF15 | ferroptosis | AKR1C3 | ferroptosis | ACSL4 | ferroptosis | SIRT1 | ferroptosis |
| TUBE1 | ferroptosis | RB1 | ferroptosis | LPCAT3 | ferroptosis | FBXW7 | ferroptosis |
| ARRDC3 | ferroptosis | HSF1 | ferroptosis | NRAS | ferroptosis | PANX1 | ferroptosis |
| CEBPG | ferroptosis | GCLC | ferroptosis | KRAS | ferroptosis | DNAJB6 | ferroptosis |
| SNORA16A | ferroptosis | SQSTM1 | ferroptosis | HRAS | ferroptosis | BACH1 | ferroptosis |
| RGS4 | ferroptosis | NQO1 | ferroptosis | TFR2 | ferroptosis | LONP1 | ferroptosis |
| MT1G | ferroptosis | MUC1 | ferroptosis | SLC38A1 | ferroptosis | SLC1A5 | ferroptosis |
