# Supplementary figures and images for: Cuproptosis-Related Ferroptosis genes for Predicting Prognosis in kidney renal clear cell carcinoma
Source: Eur J Med Res. 2023 May 15;28:176. doi: 10.1186/s40001-023-01137-z (PMC10184413; doi:10.1186/s40001-023-01137-z)

A

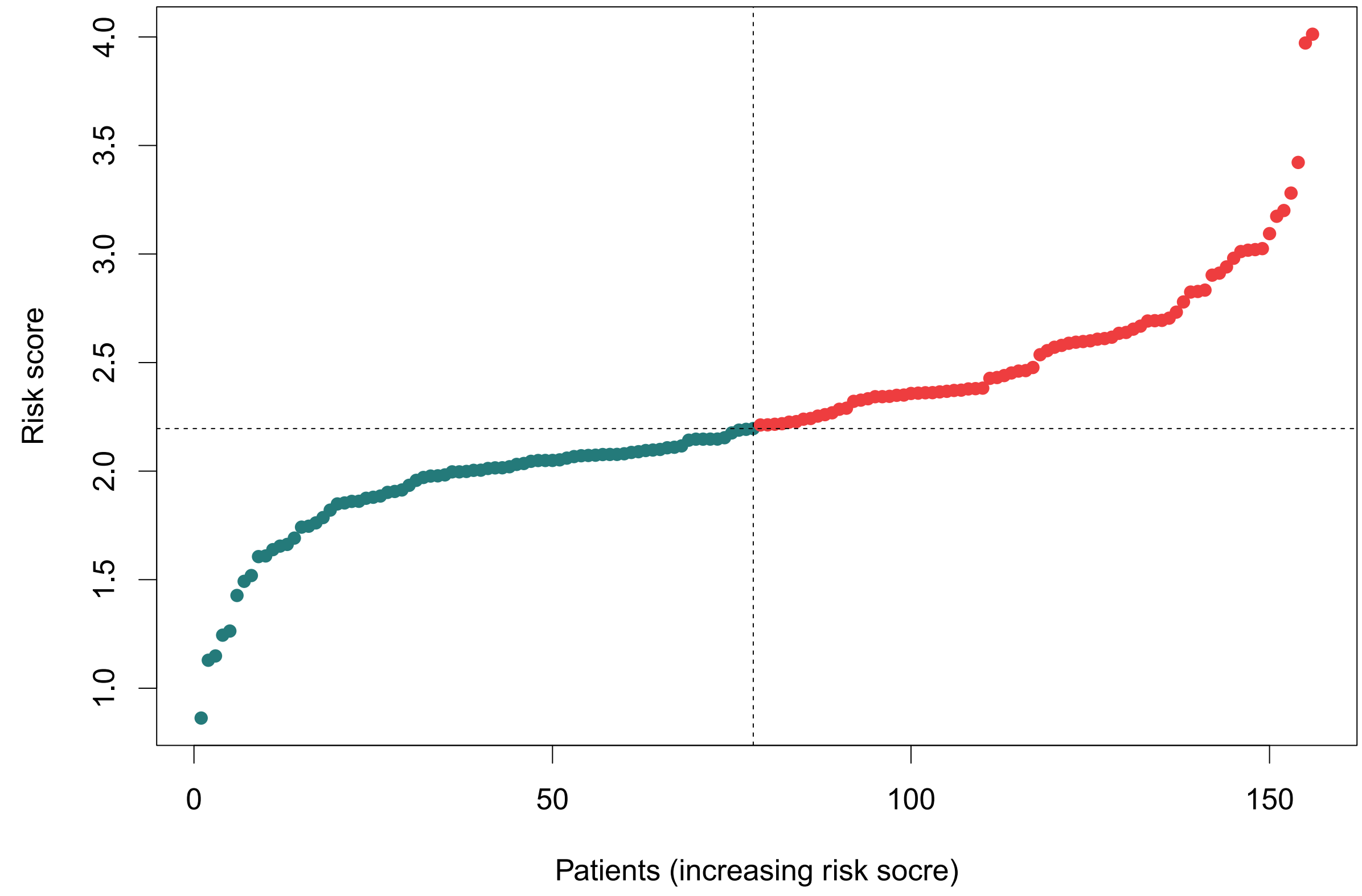

B

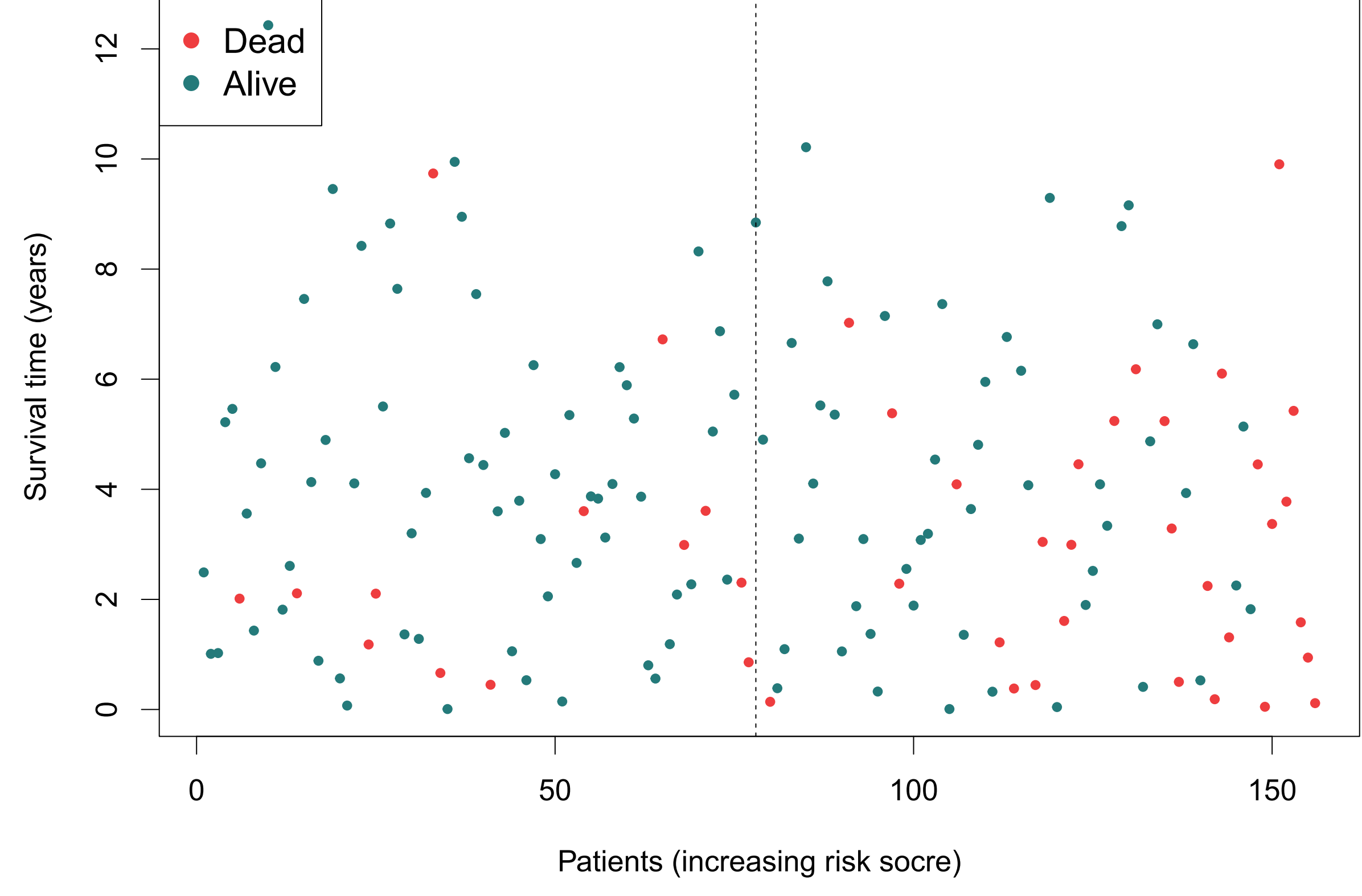

C

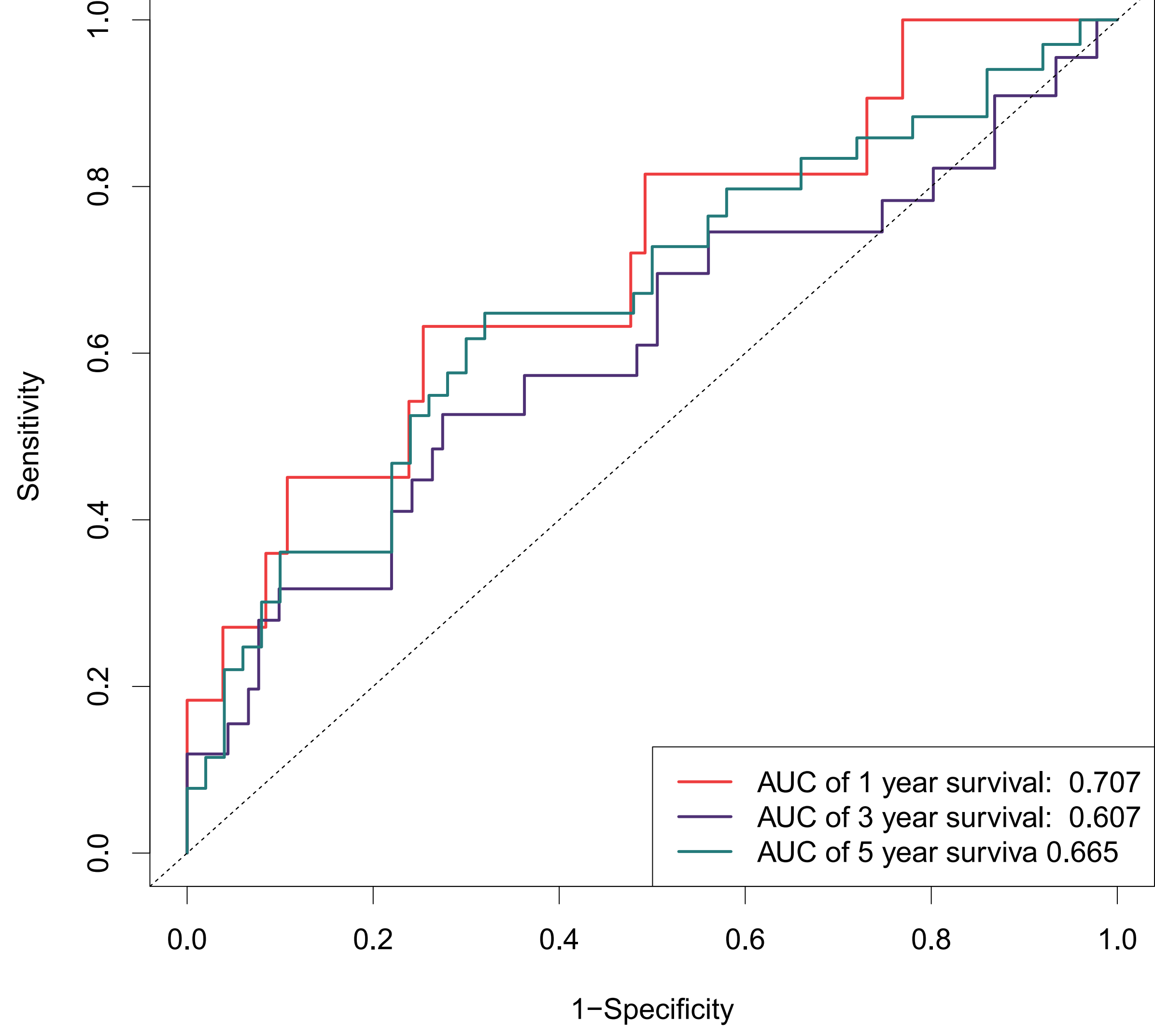

D

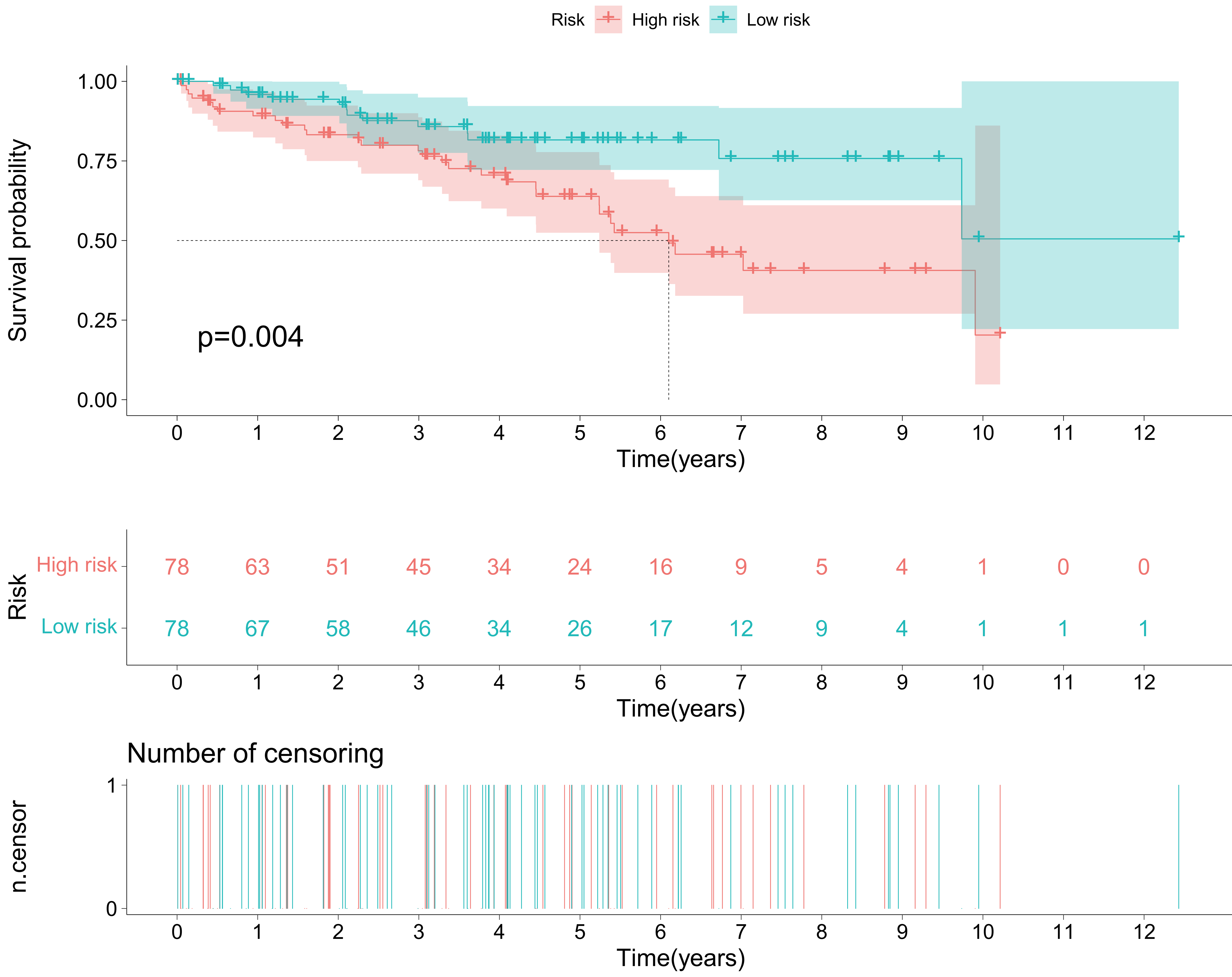

Supplement: Supplementary file 2 — Additional file 2: Figure S1.Distribution of survival status based on the median risk score in validation set;The receiver operating characteristiccurve analyses of the prognostic CRFGs in predicting 1-, 3-, and 5-year overall survival;Kaplan–Meier survival analysis of KIRC patients between high-risk groups and low-risk groups. [file 40001_2023_1137_MOESM2_ESM.pdf]

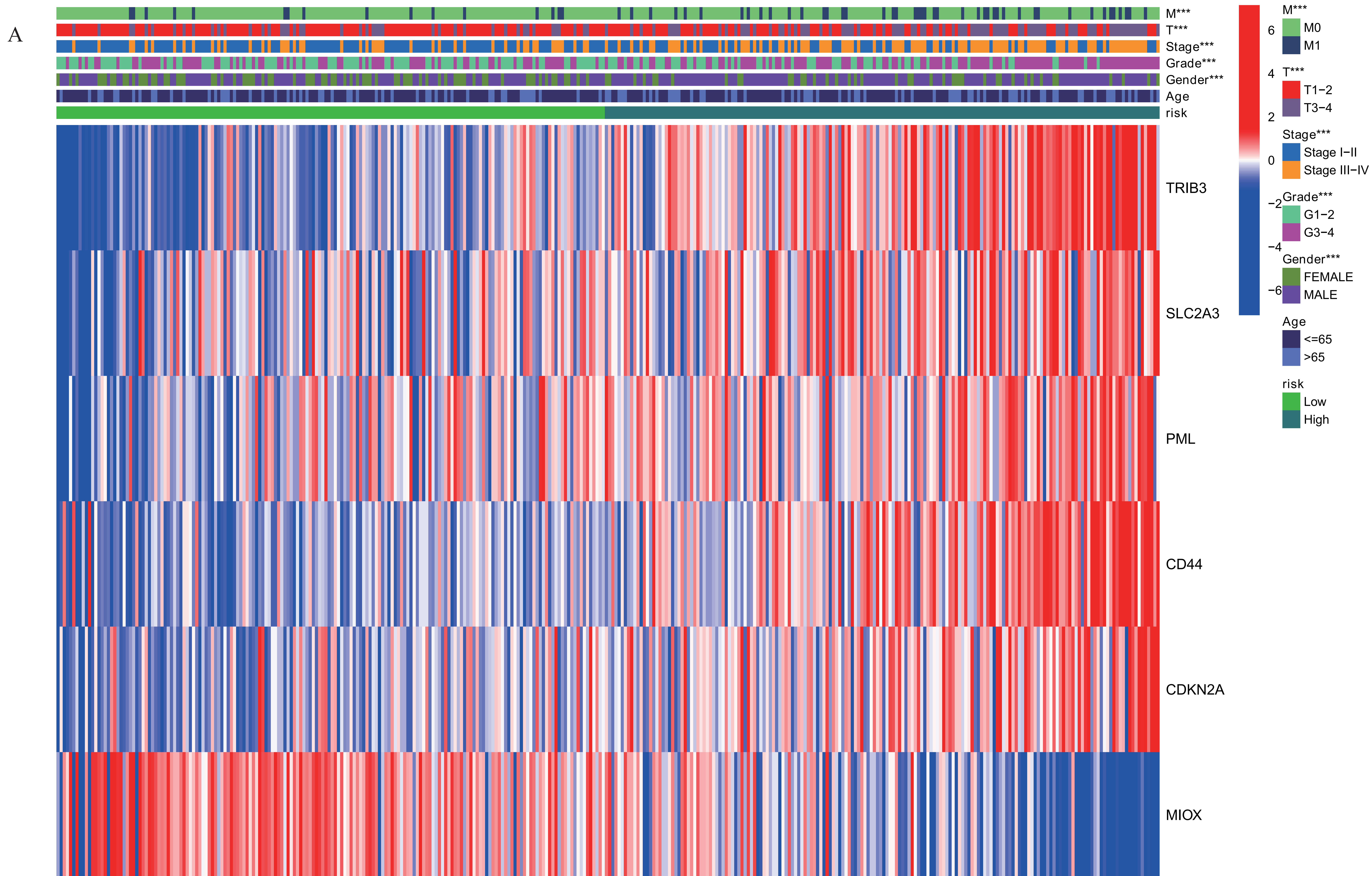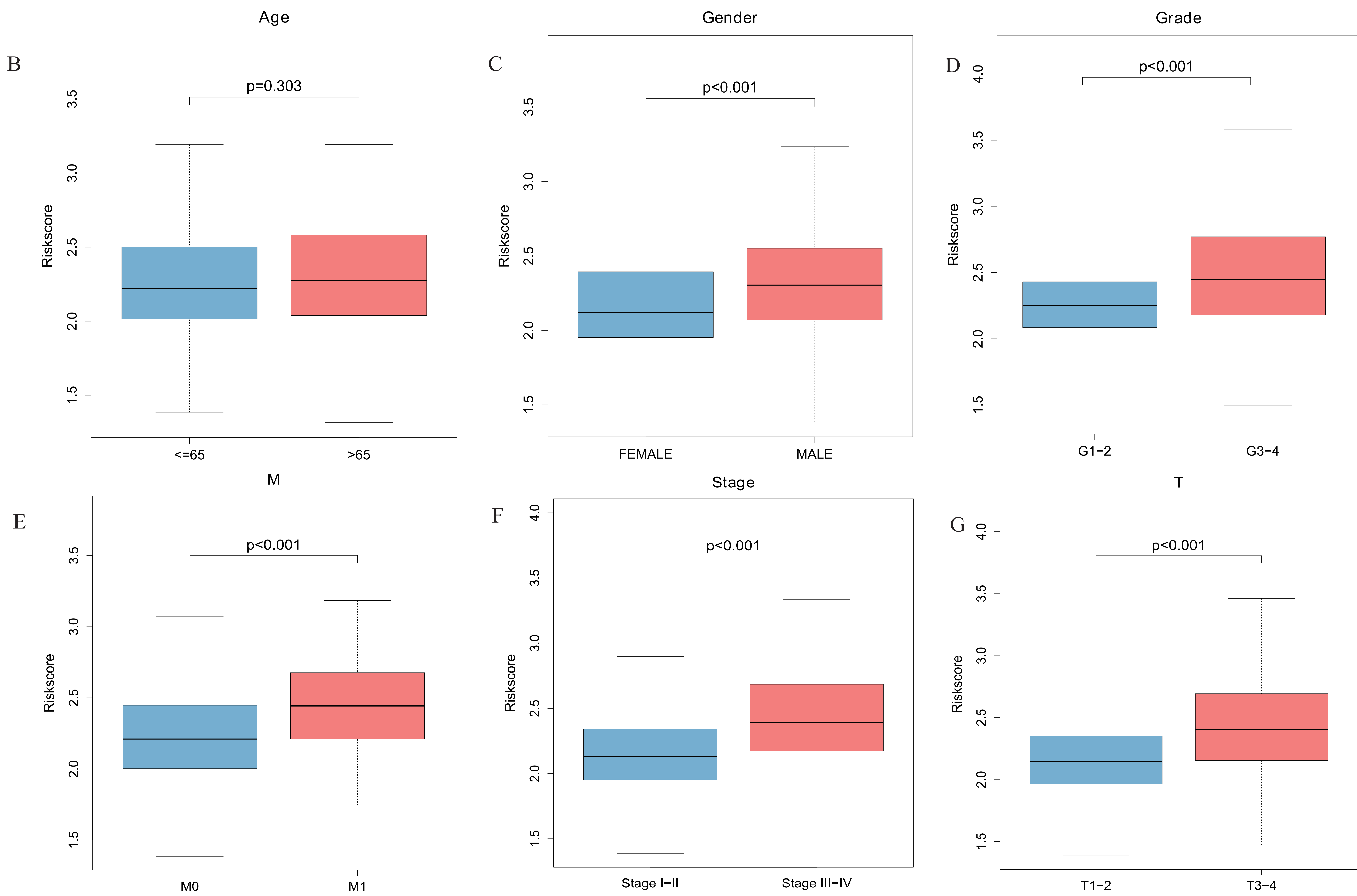

Supplement: Supplementary file 3 — Additional file 3: Figure S2. Correlation between signature and clinical characteristics. [file 40001_2023_1137_MOESM3_ESM.pdf]

A

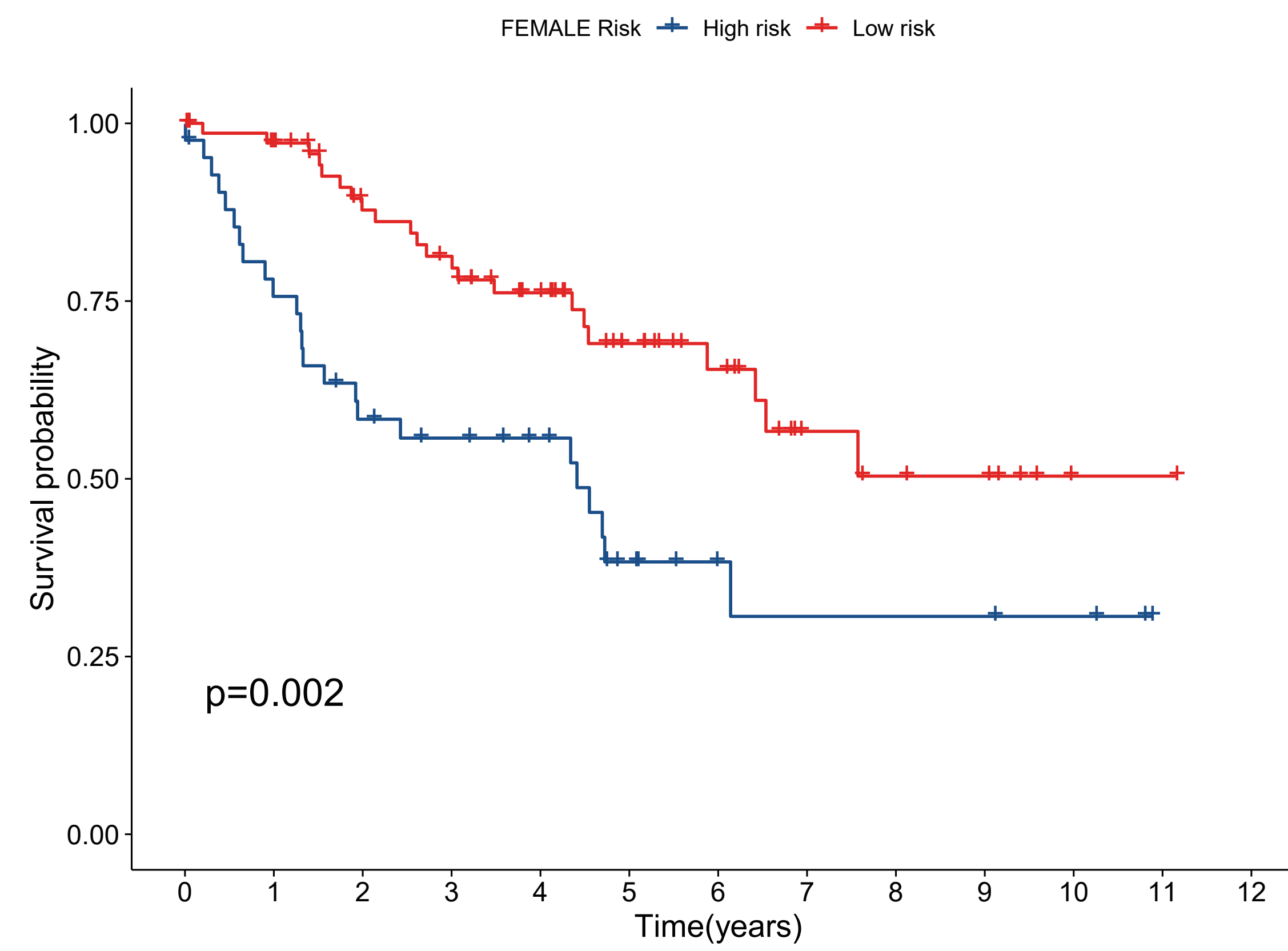

B

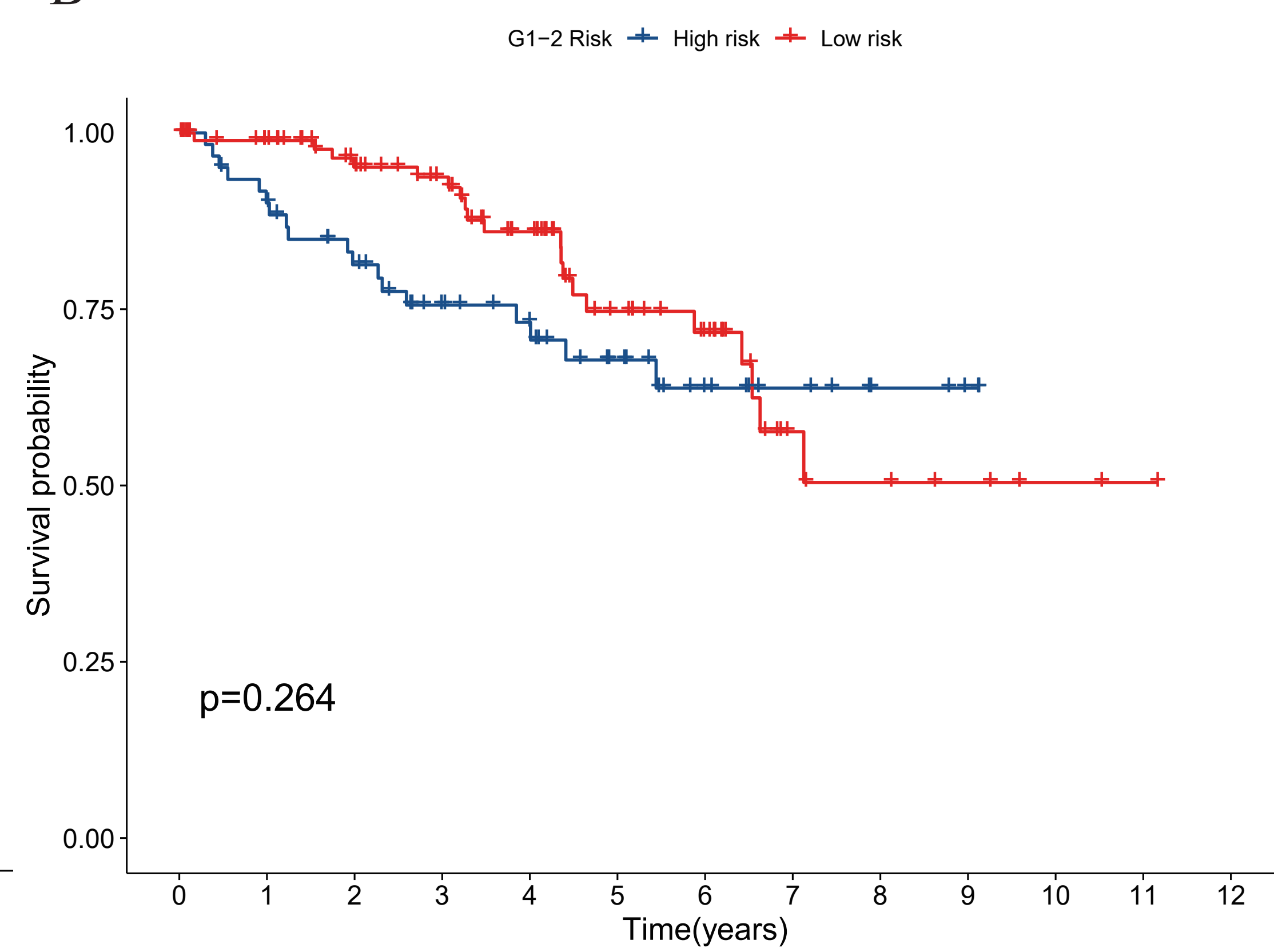

C

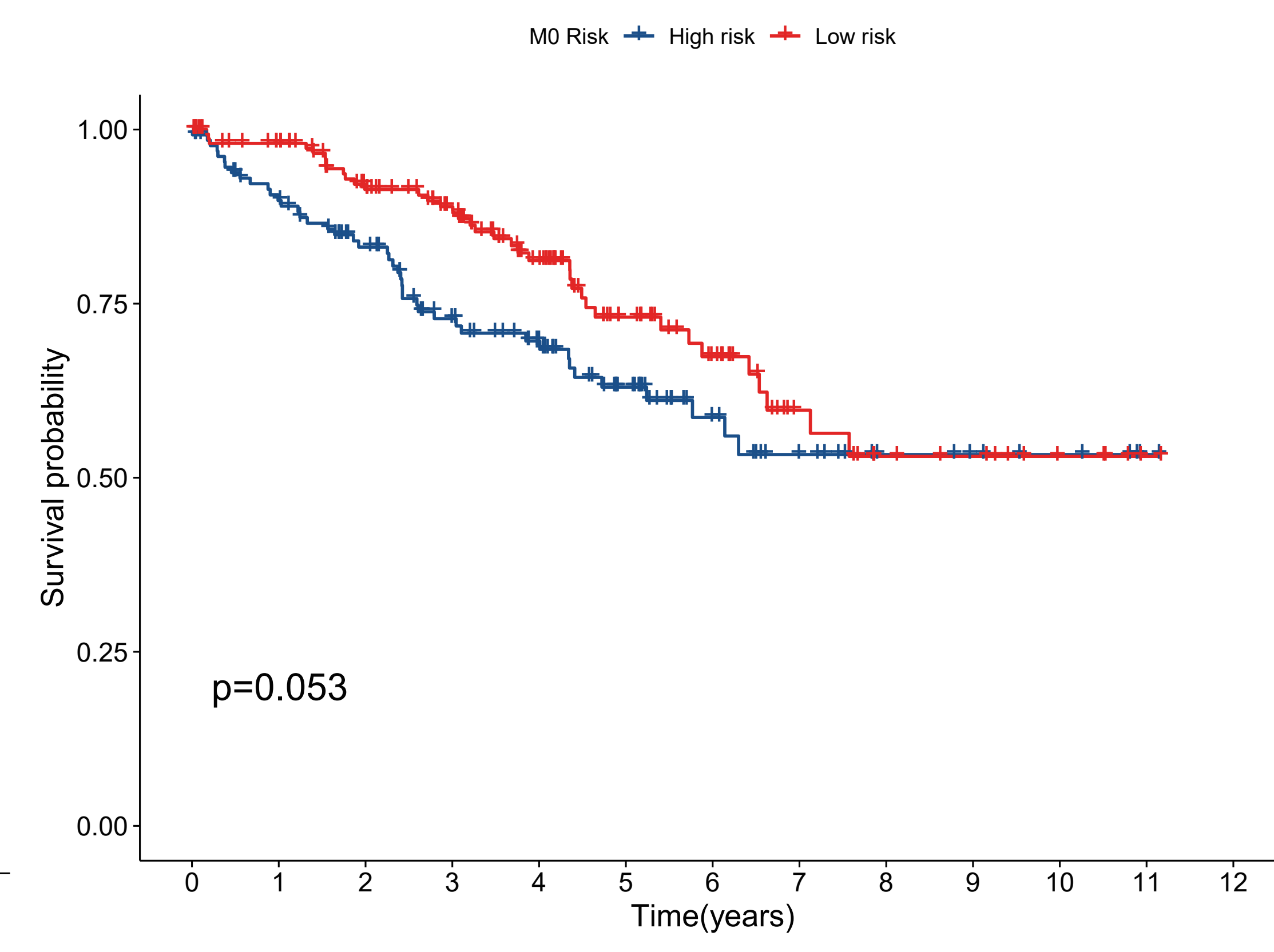

D

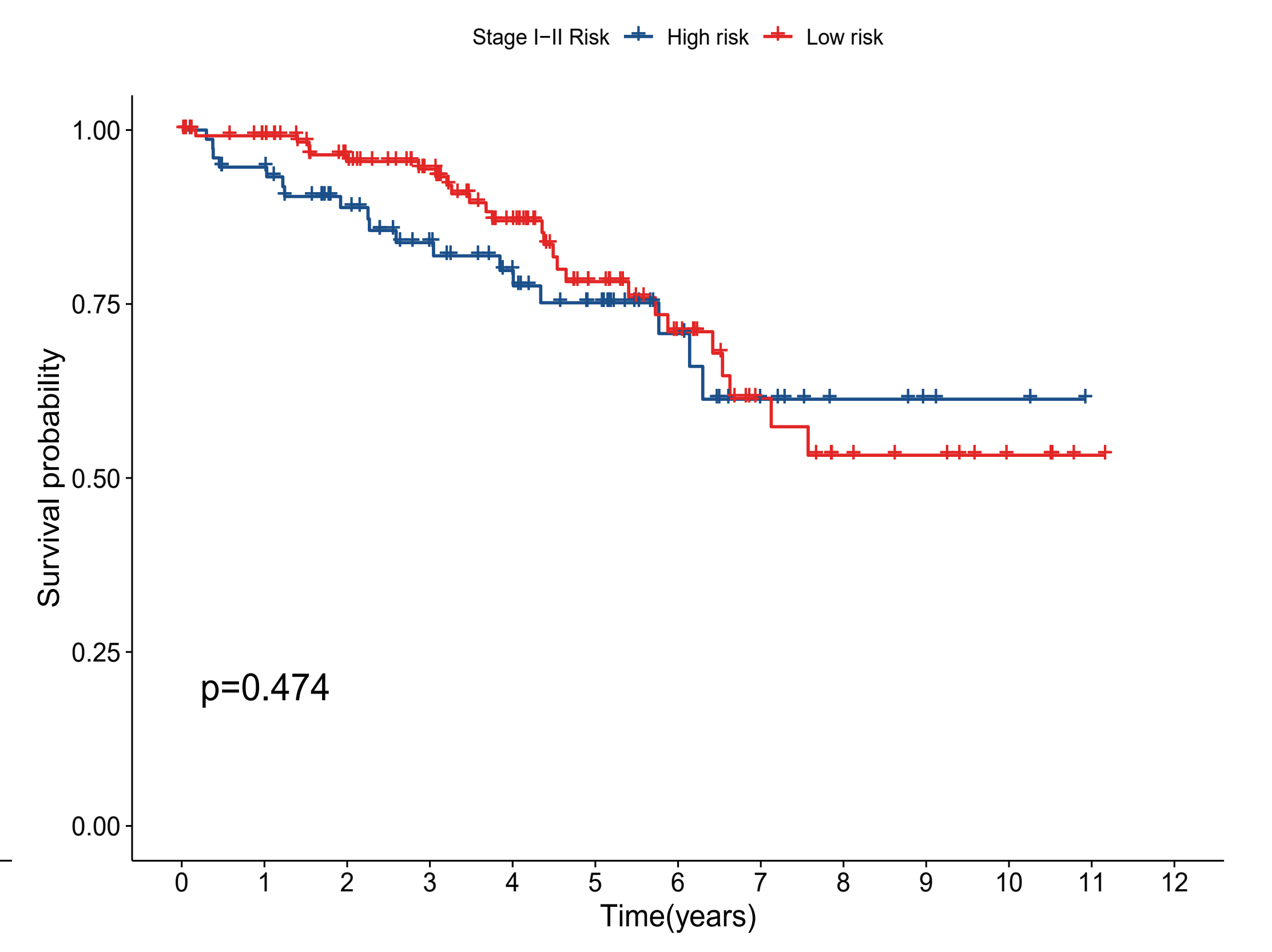

E

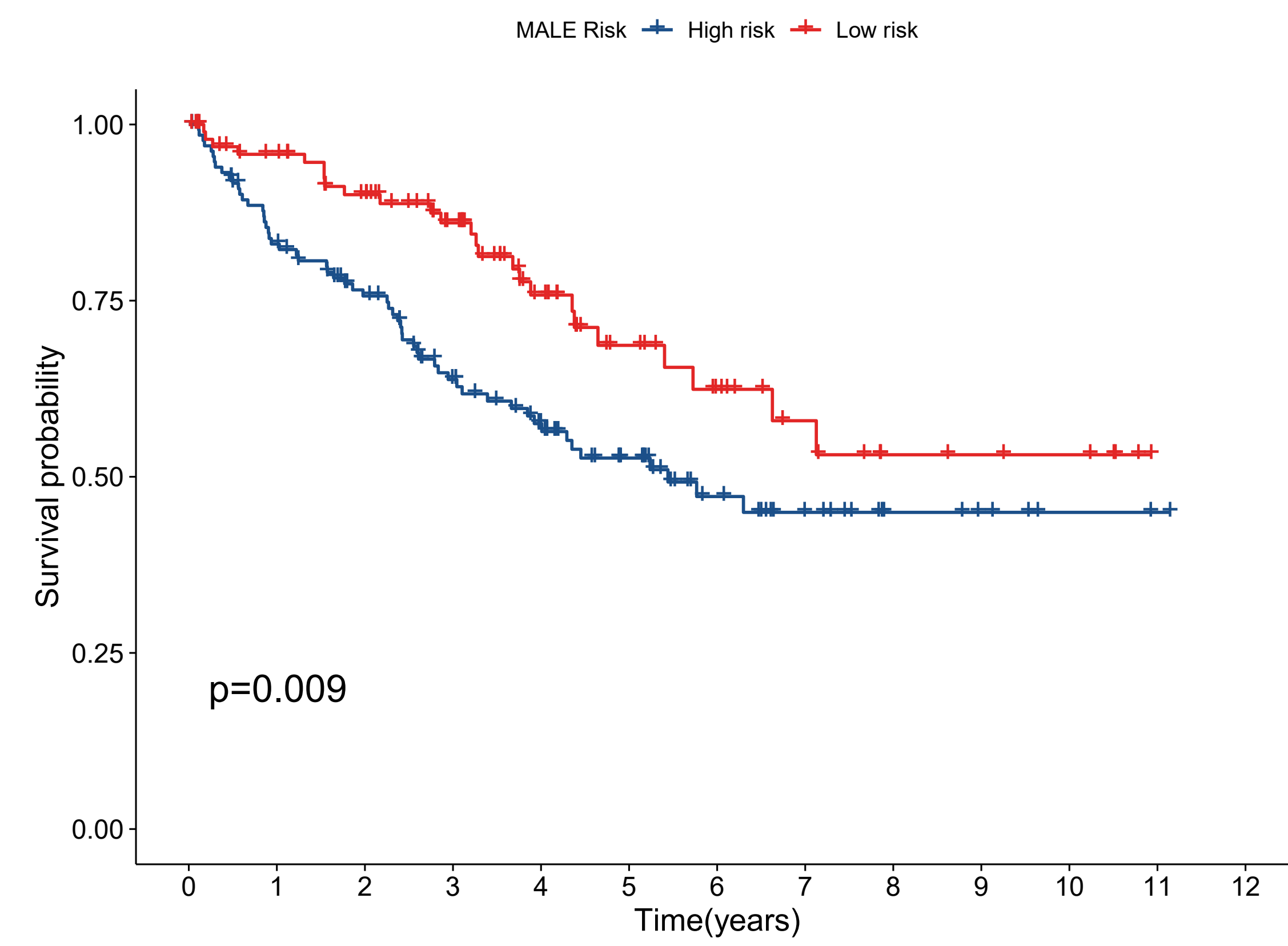

F

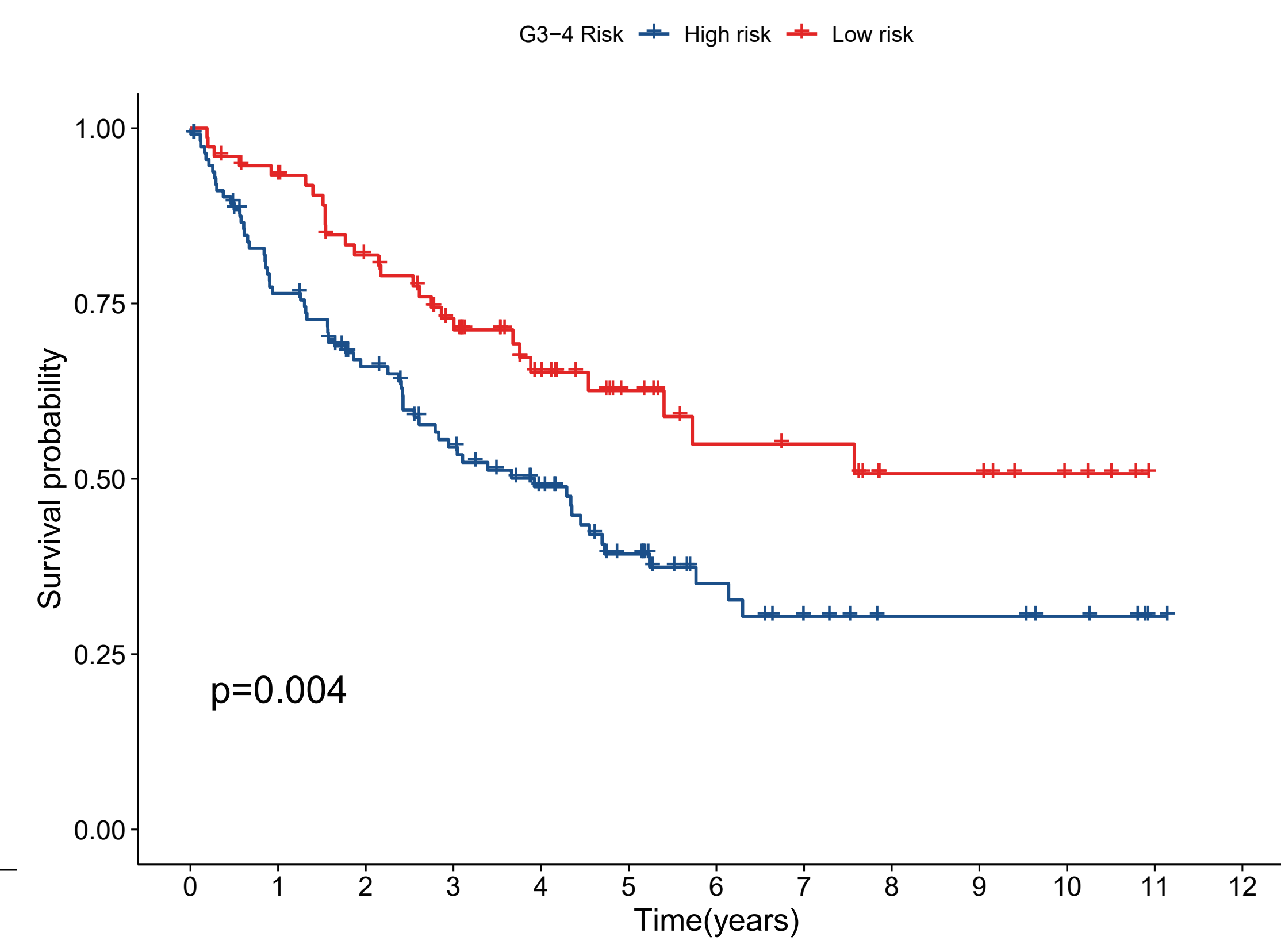

G

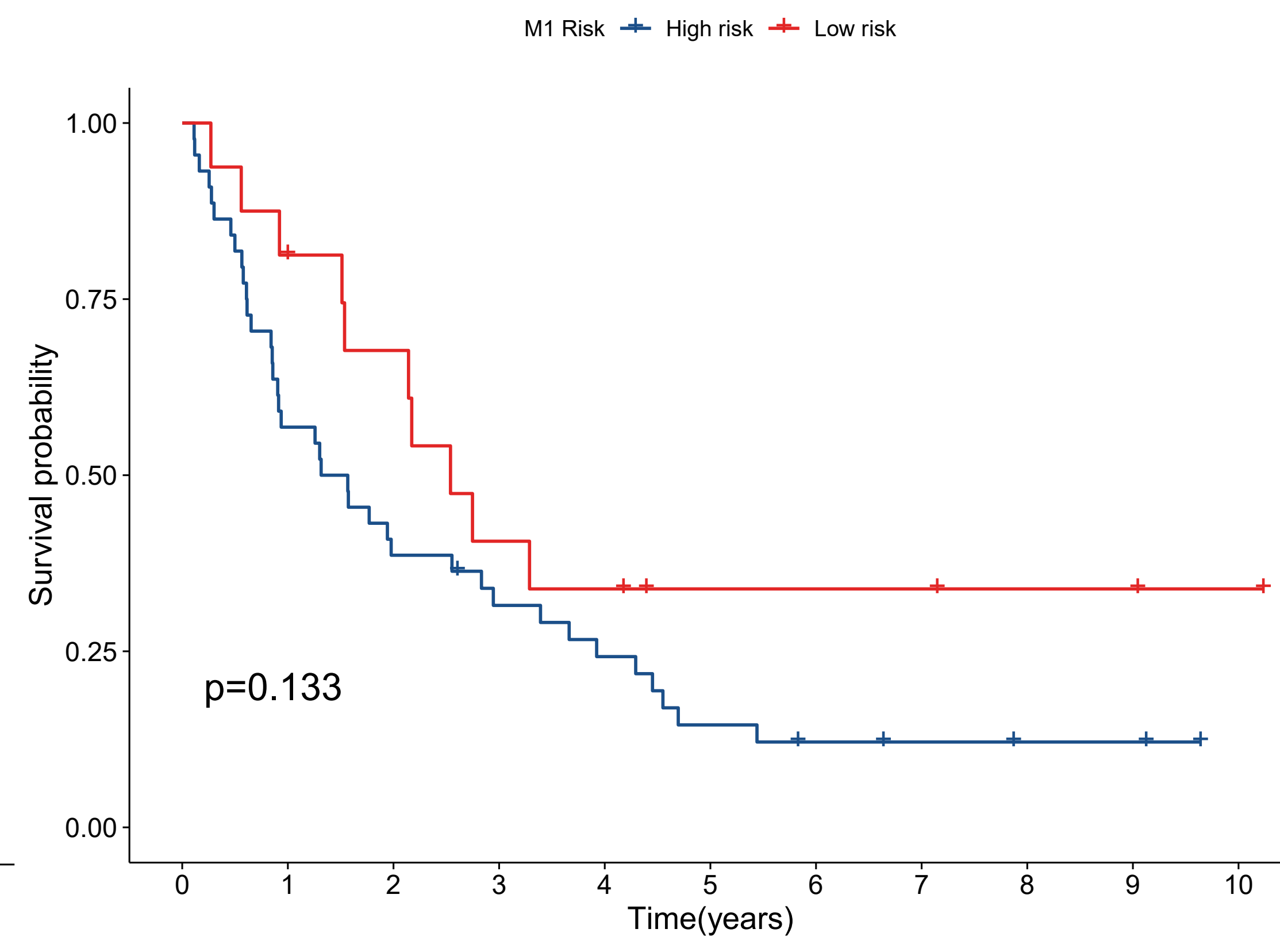

H

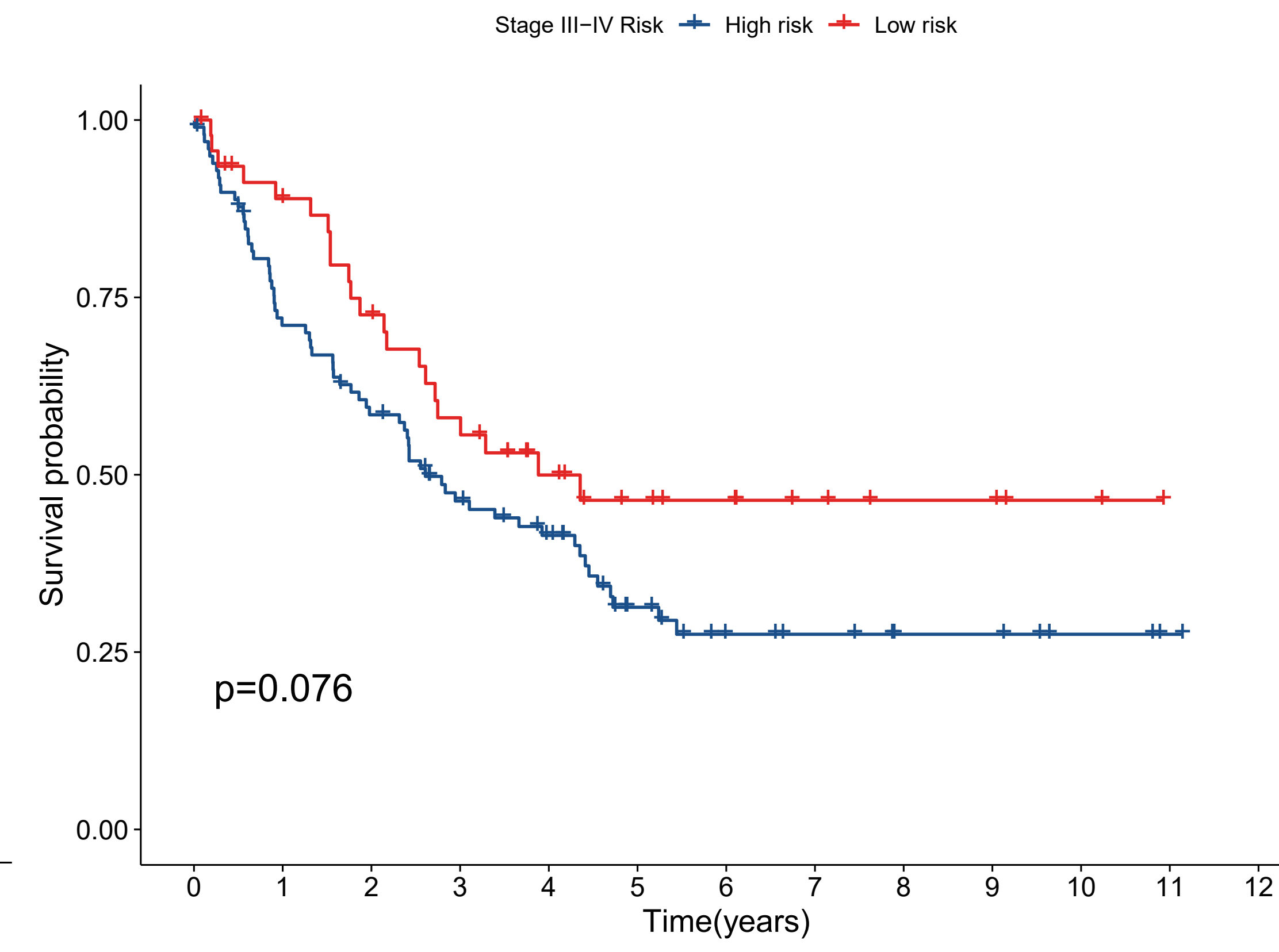

I

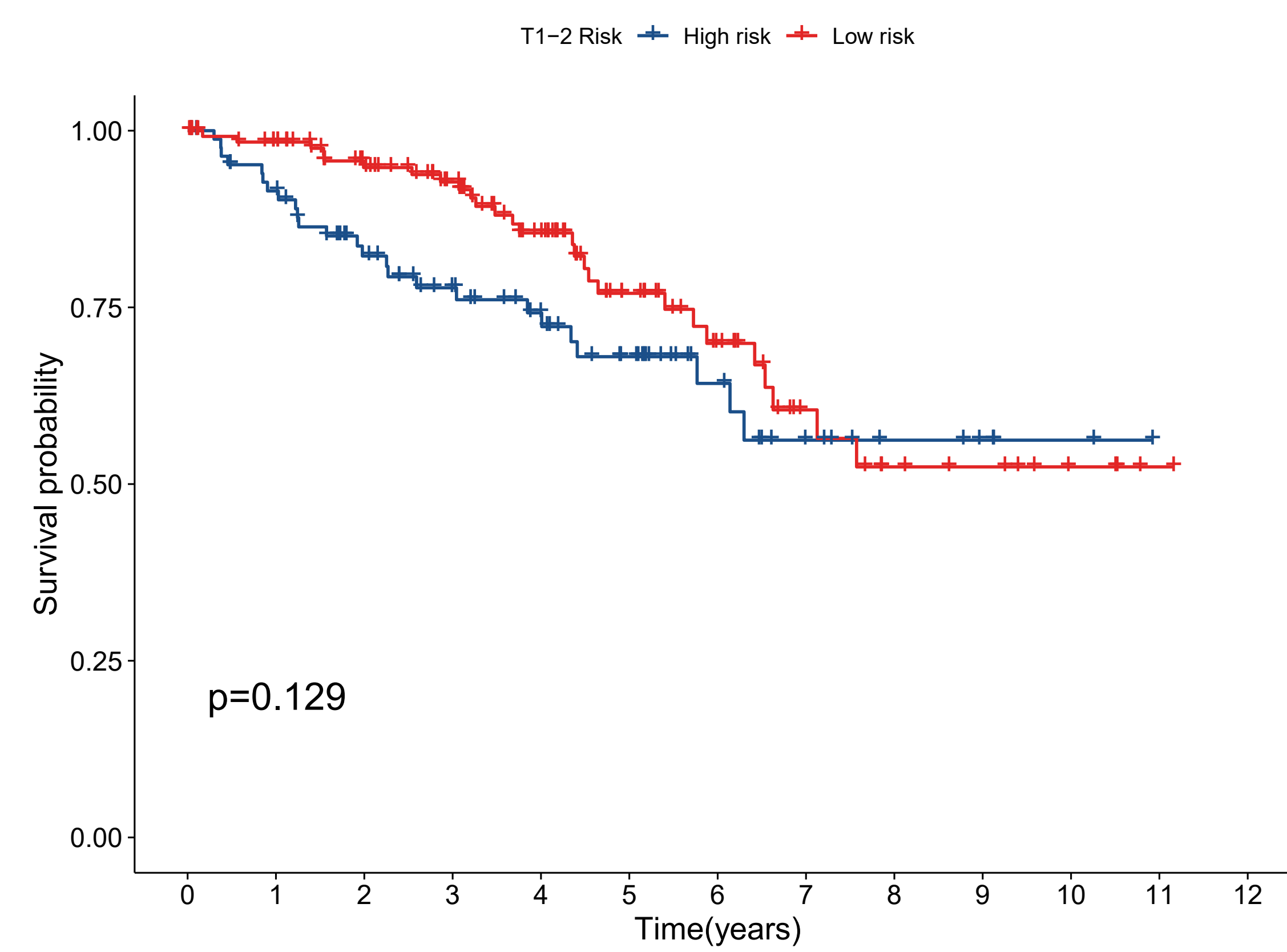

J

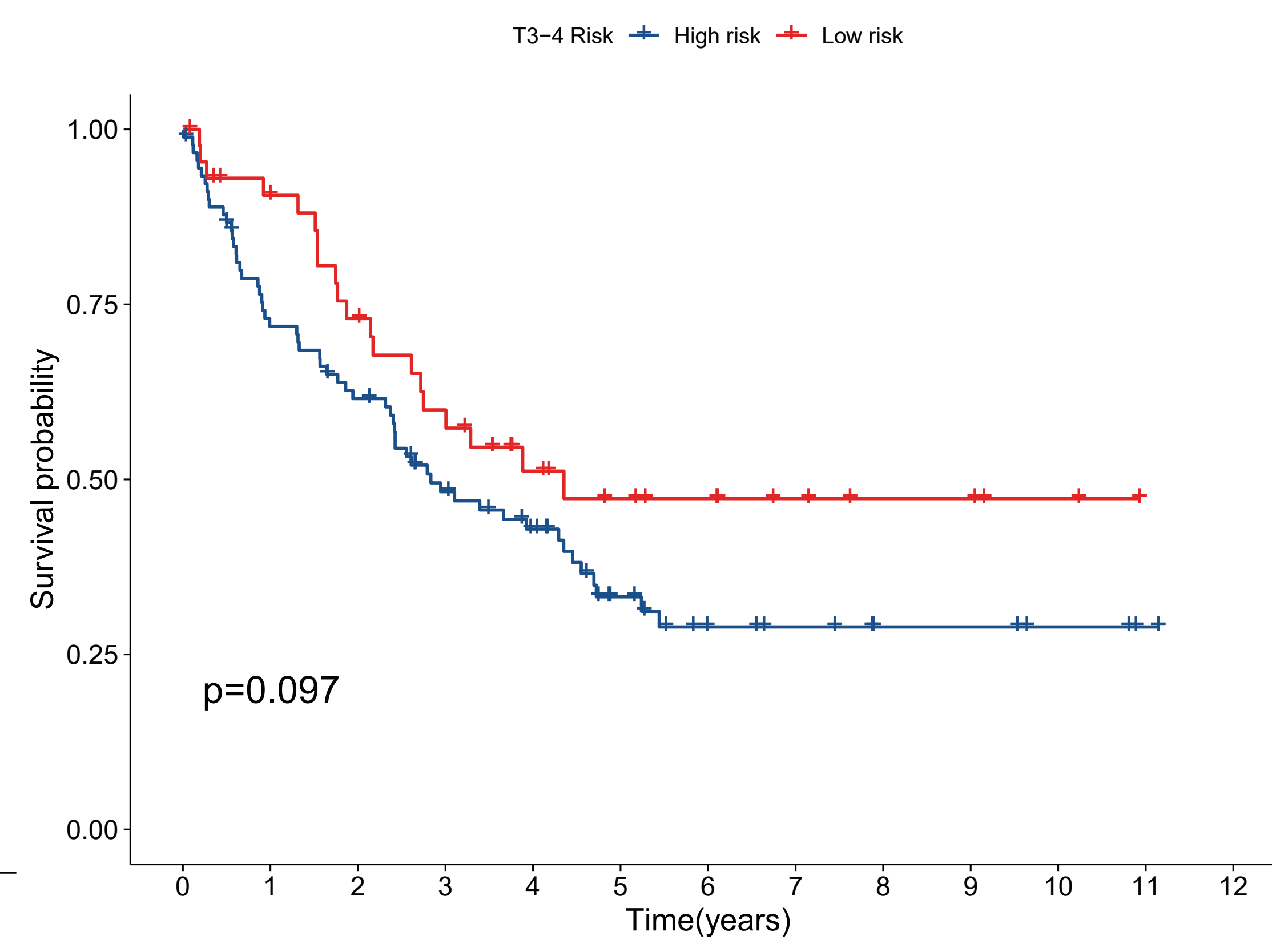

Supplement: Supplementary file 4 — Additional file 4: Figure S3. Kaplan–Meier curves of OS diferences stratifed by gender, age, grade, N stage, T stage, or M stage between the high-risk groups and low-risk groups. [file 40001_2023_1137_MOESM4_ESM.pdf]

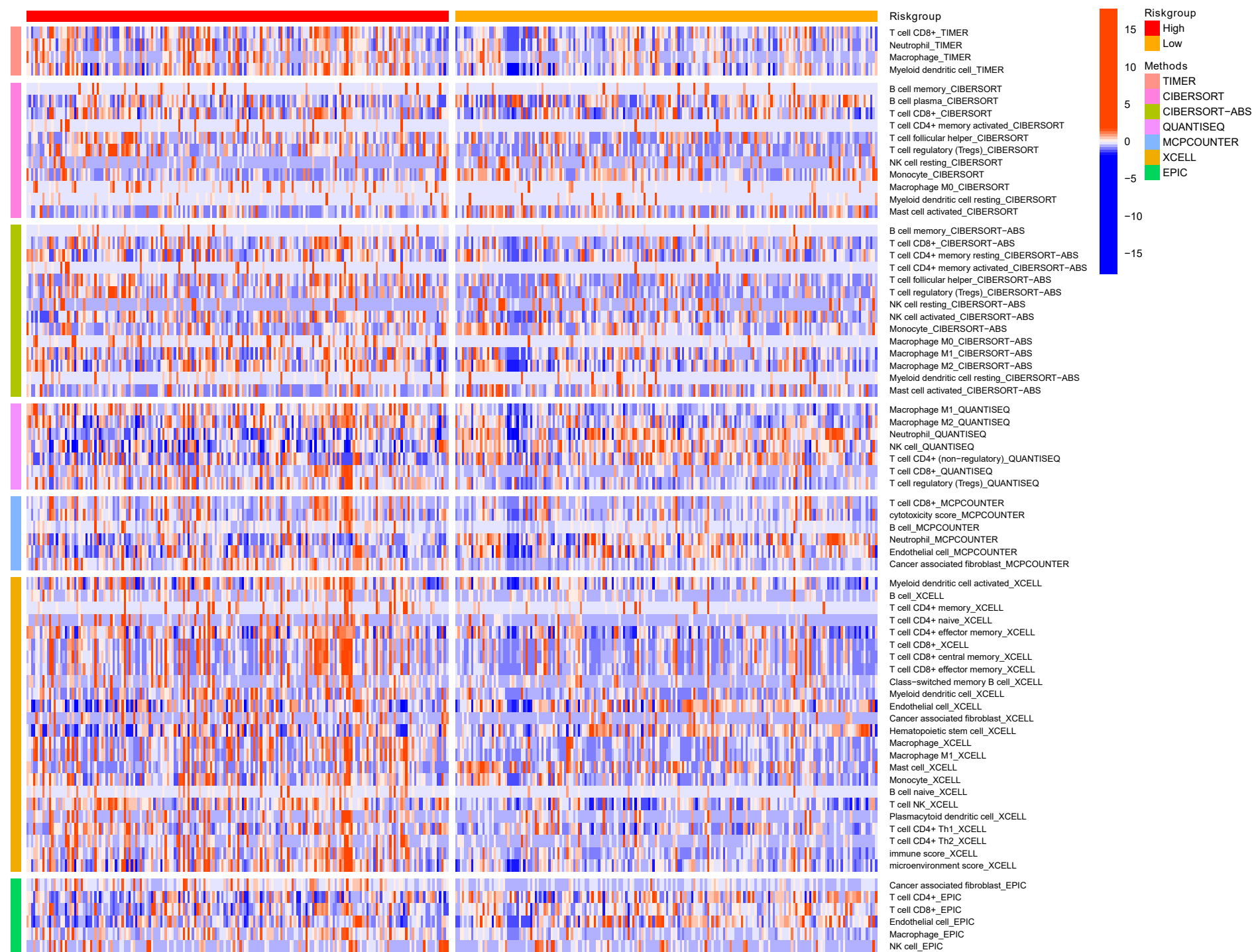

Supplement: Supplementary file 5 — Additional file 5: Figure S4. Immune cells infiltration between high-risk groups and low-risk groups. [file 40001_2023_1137_MOESM5_ESM.pdf]

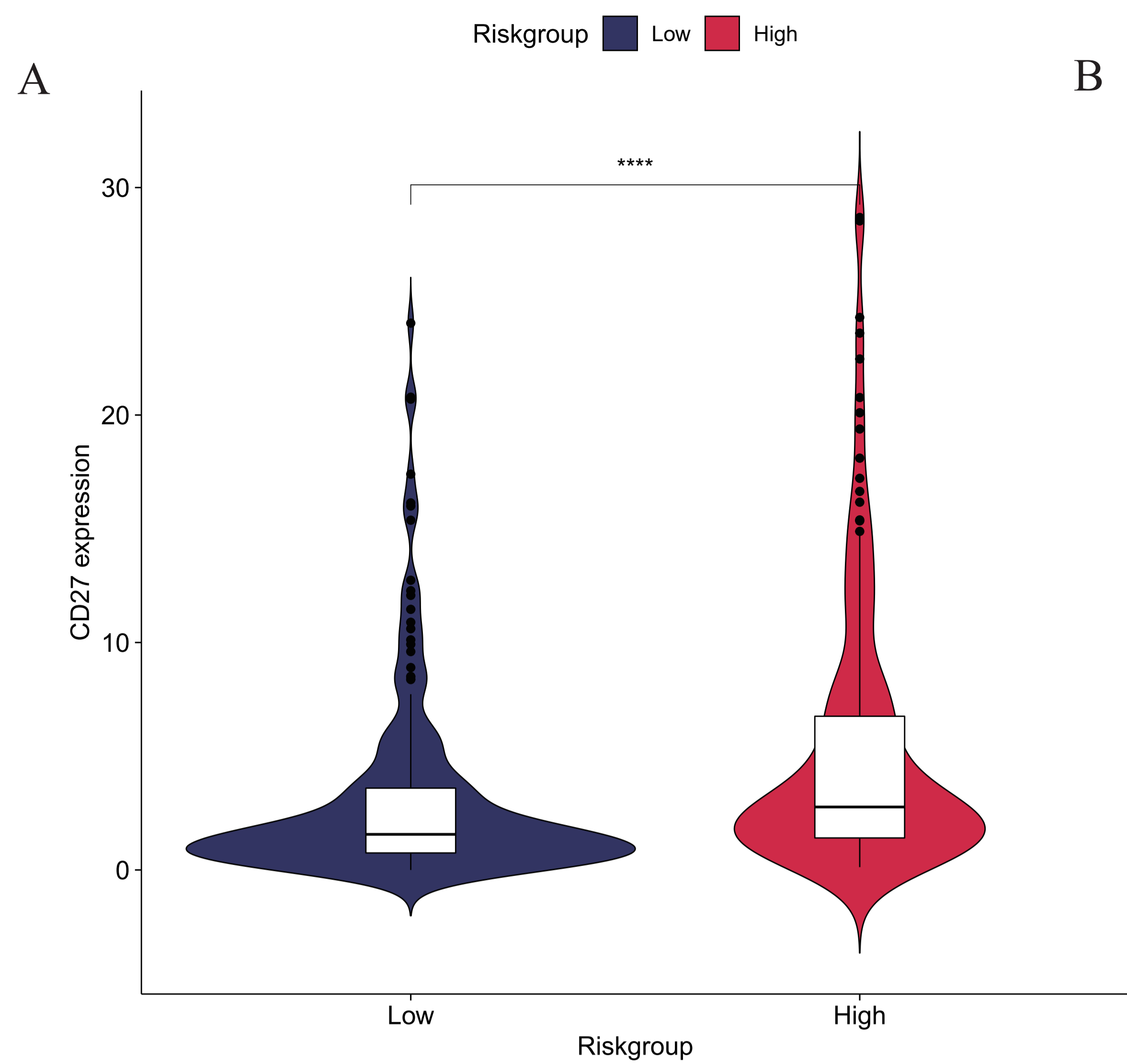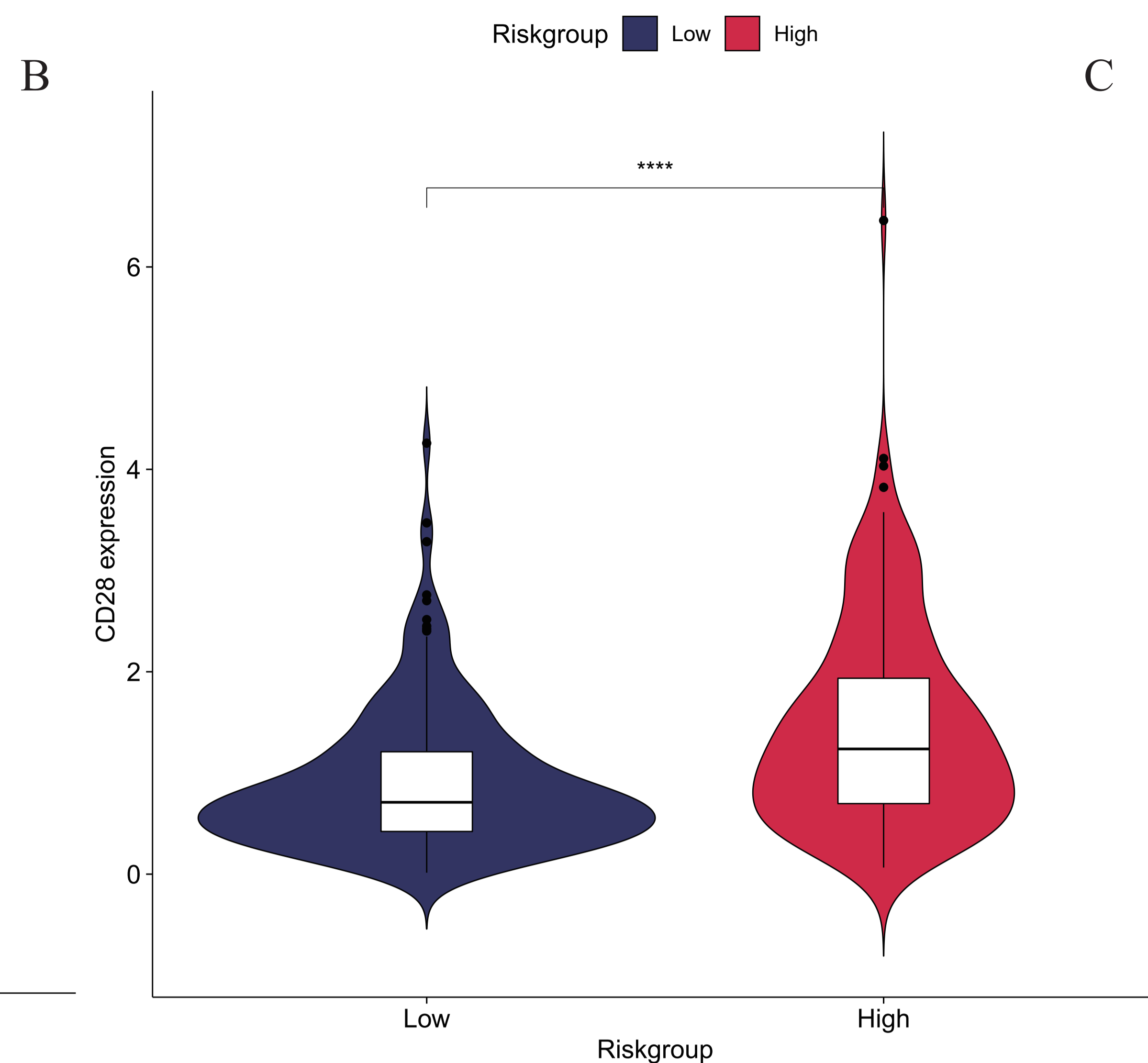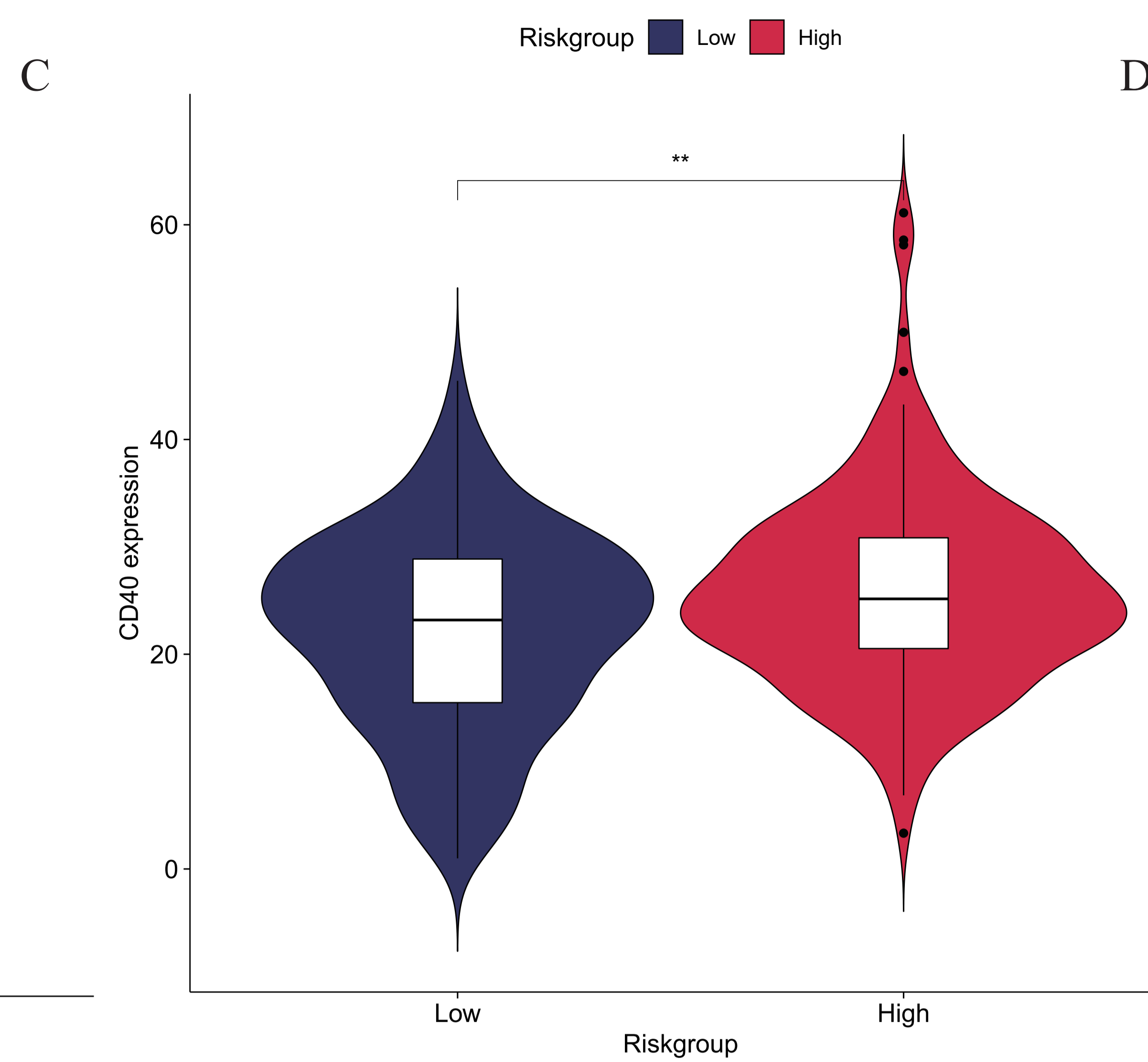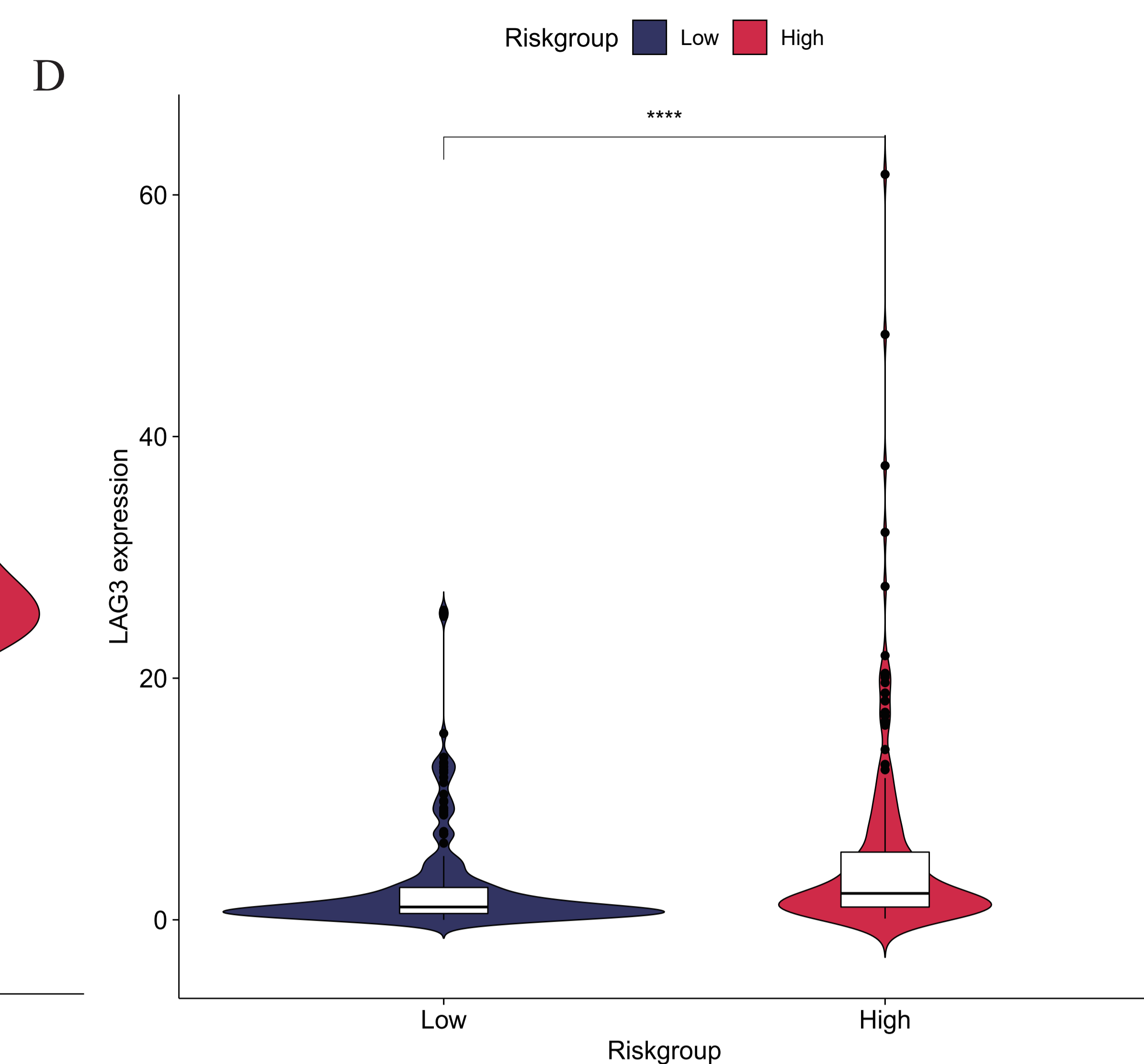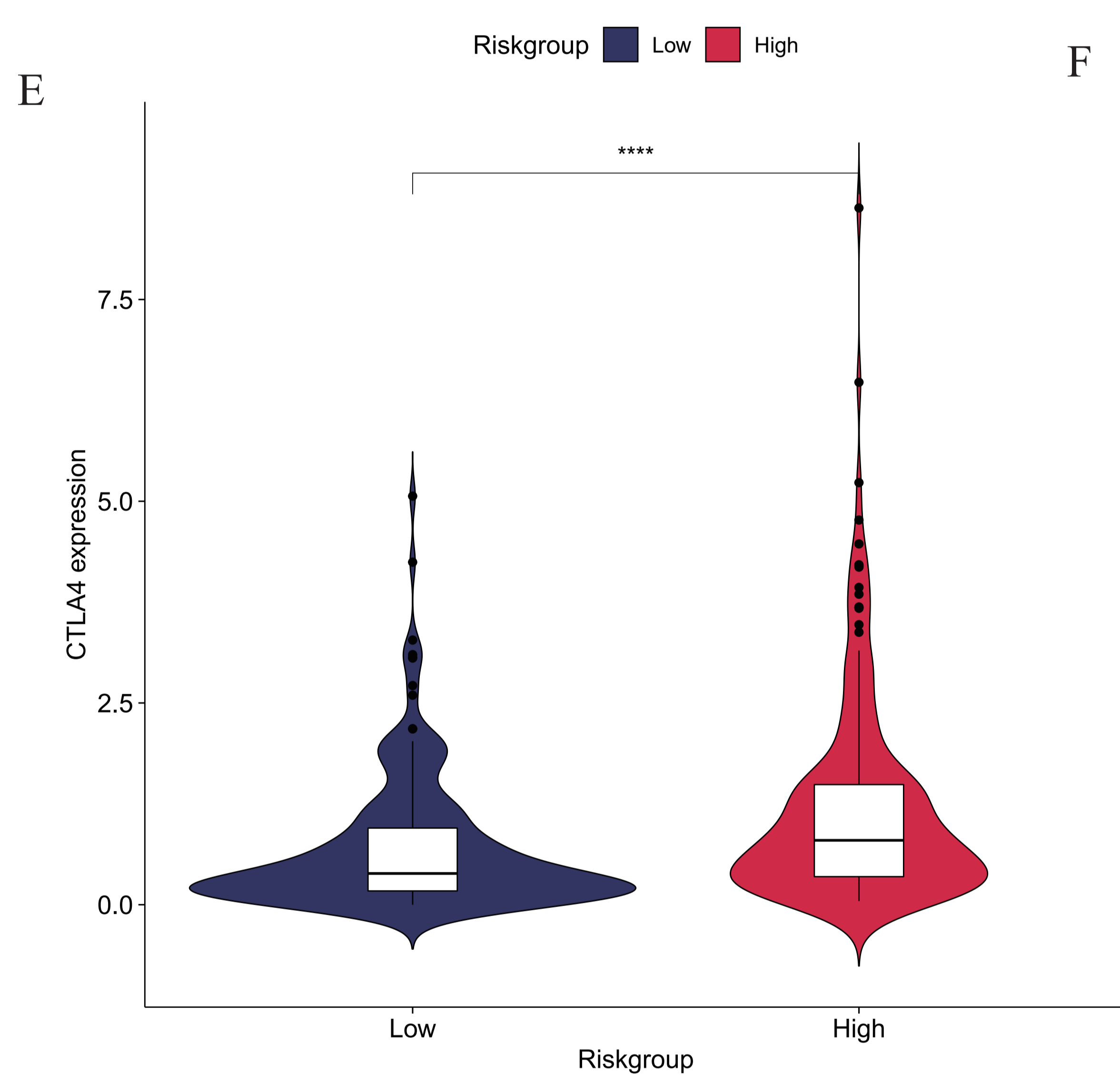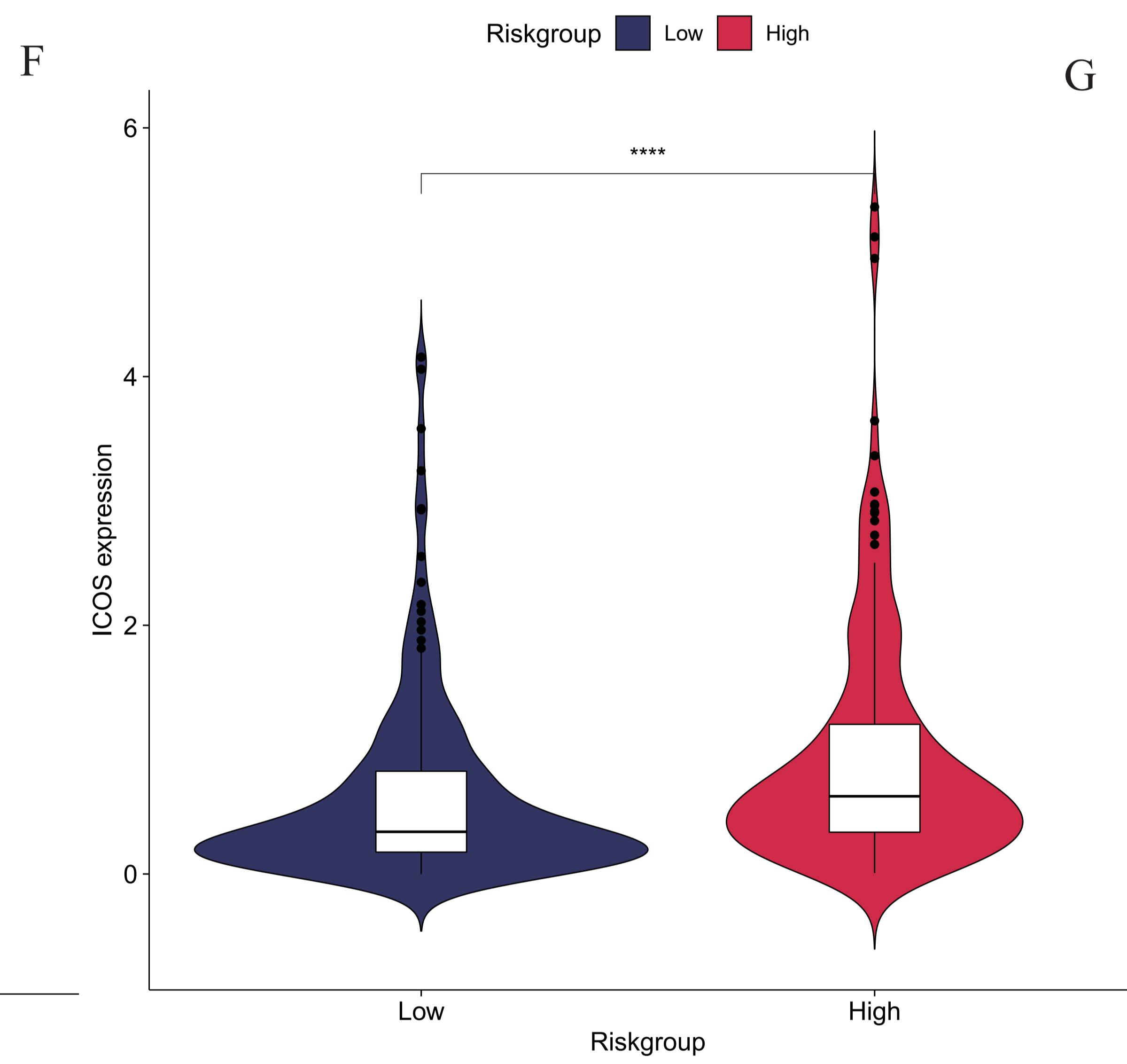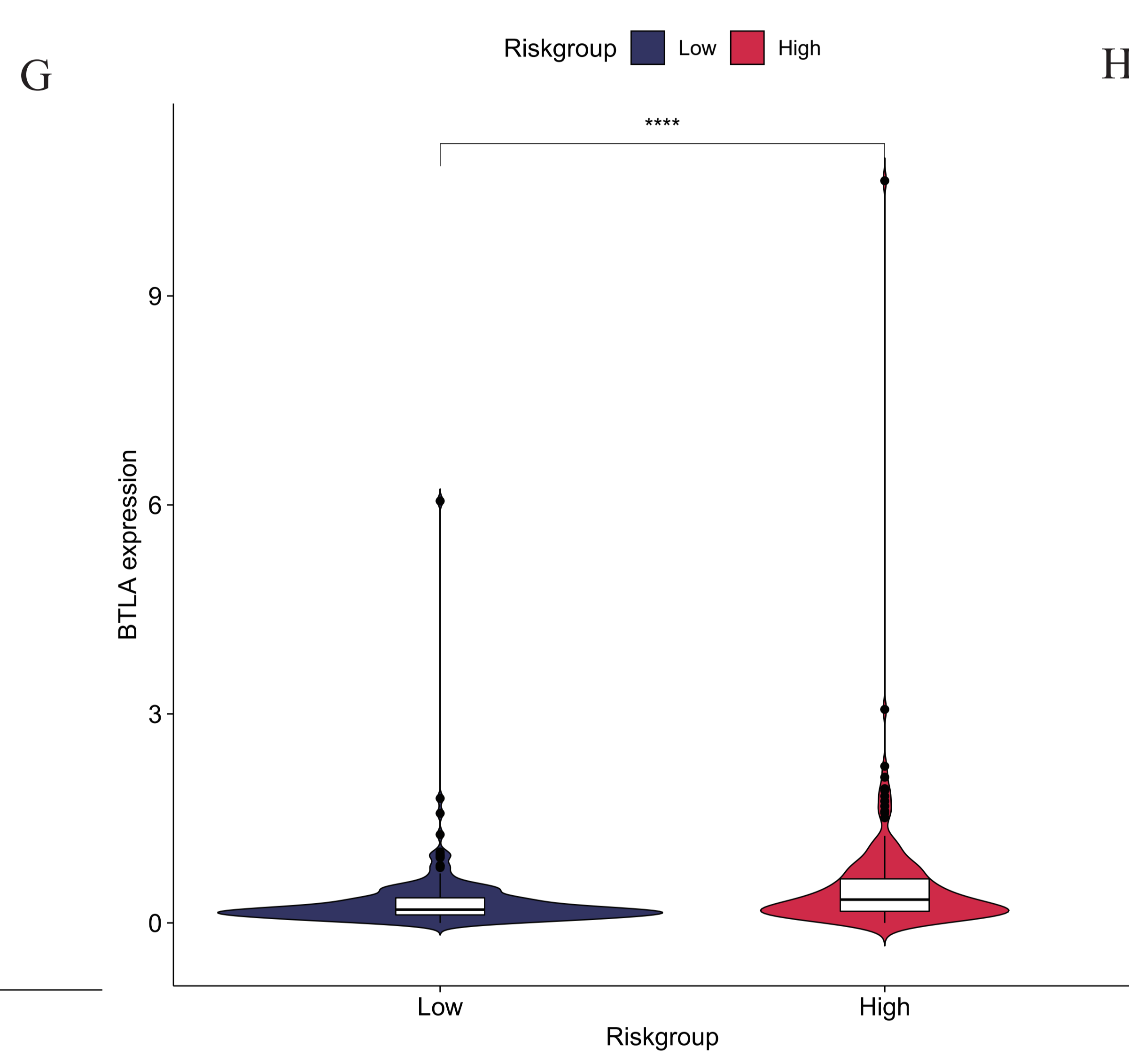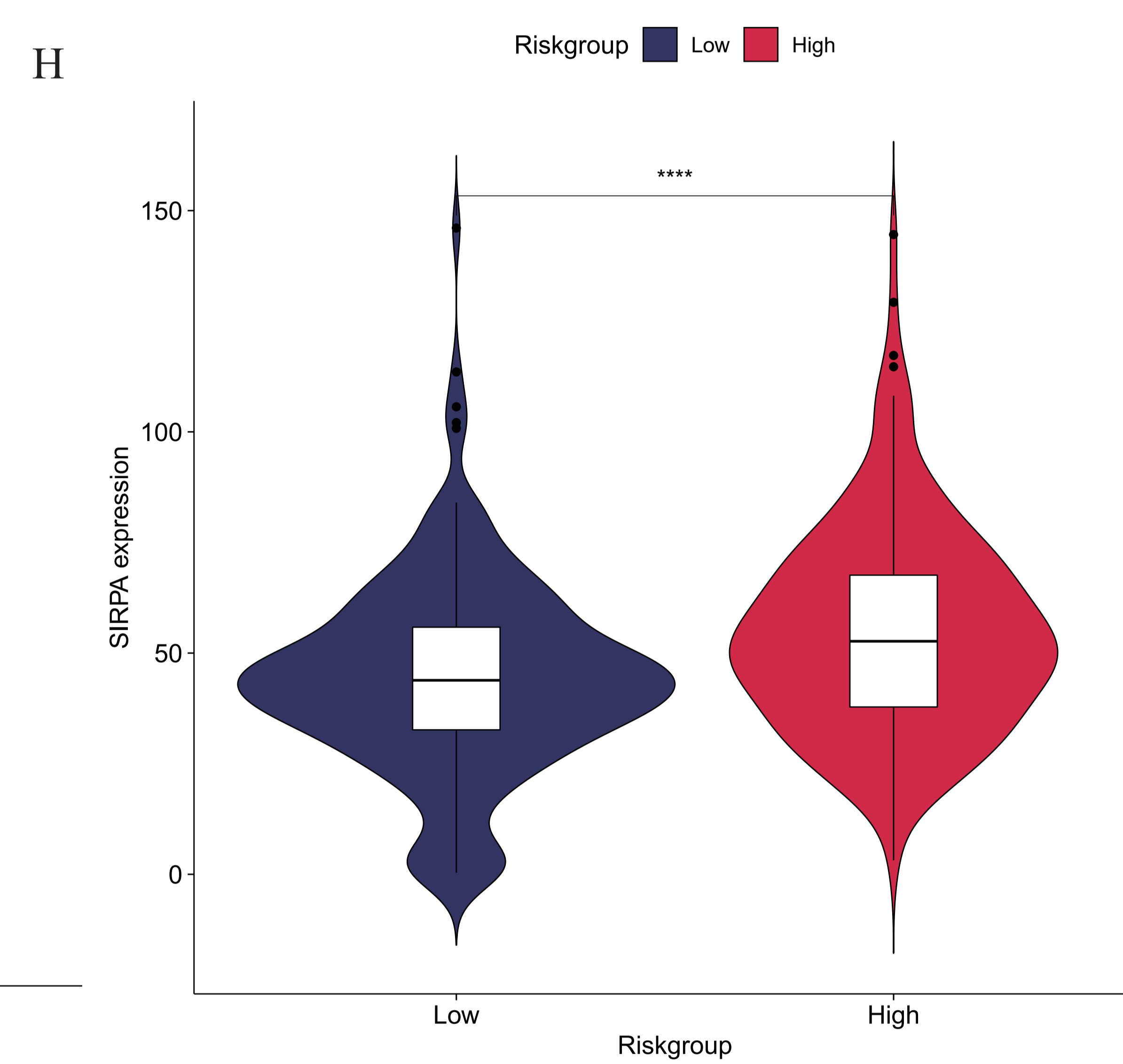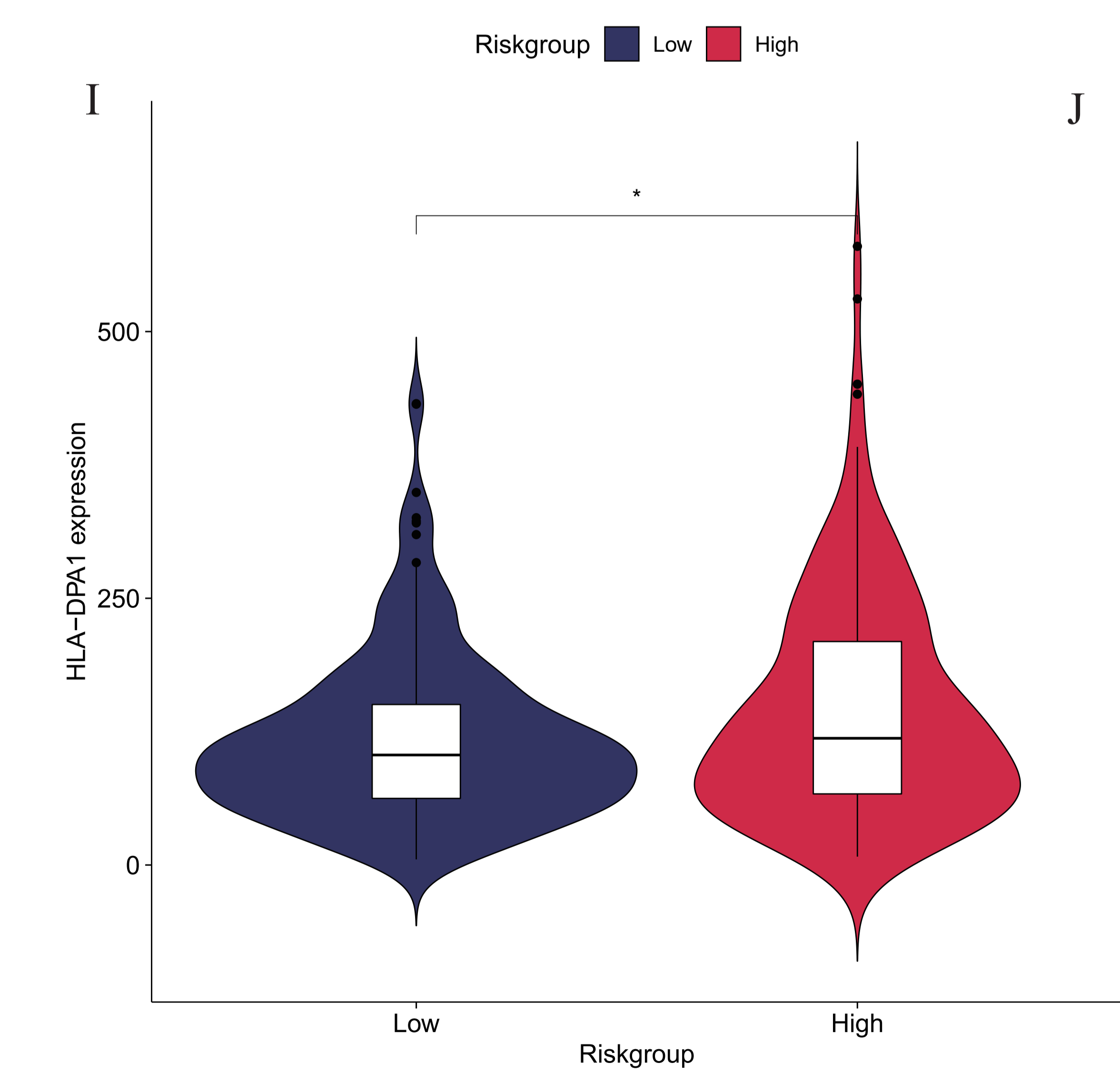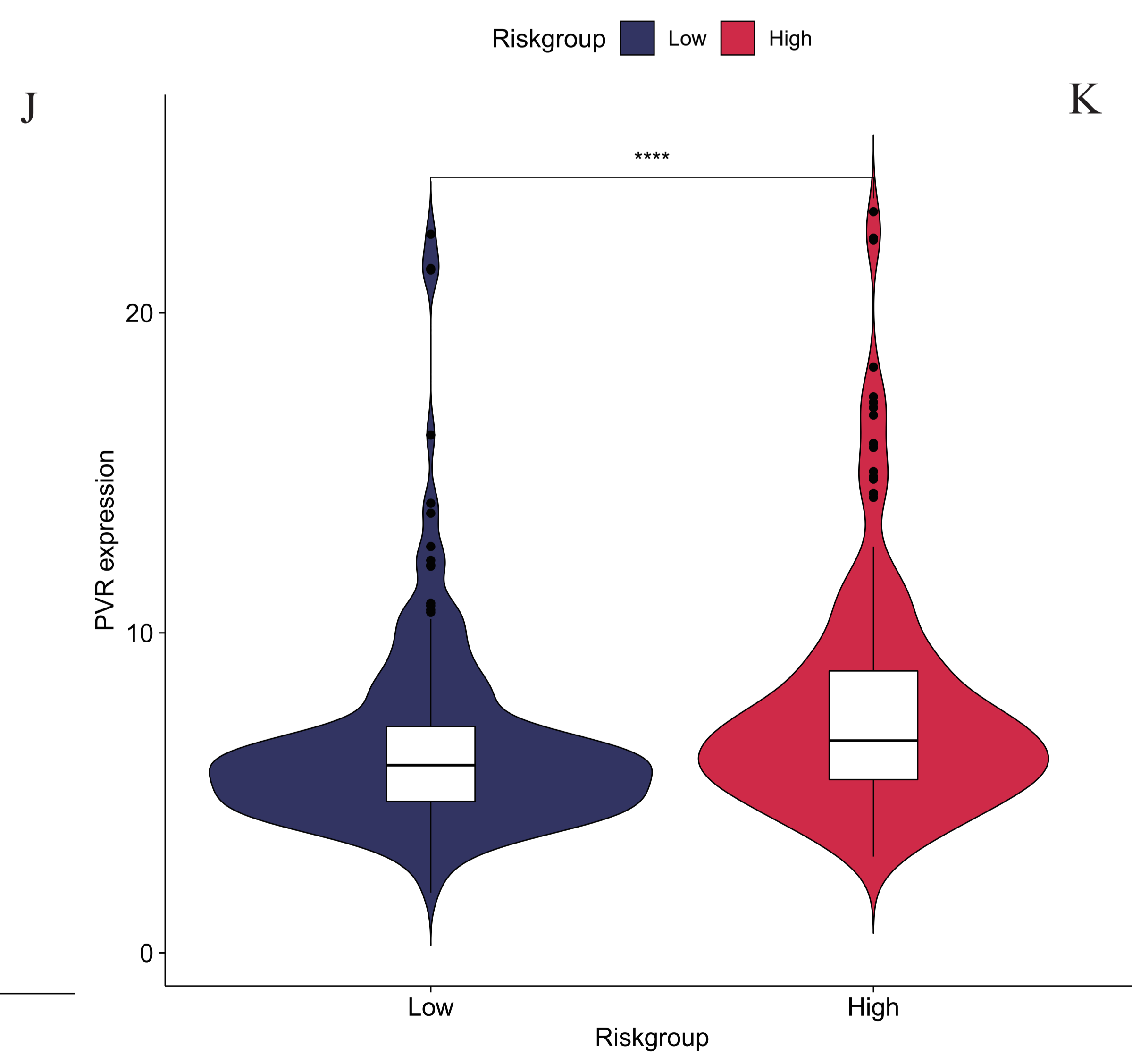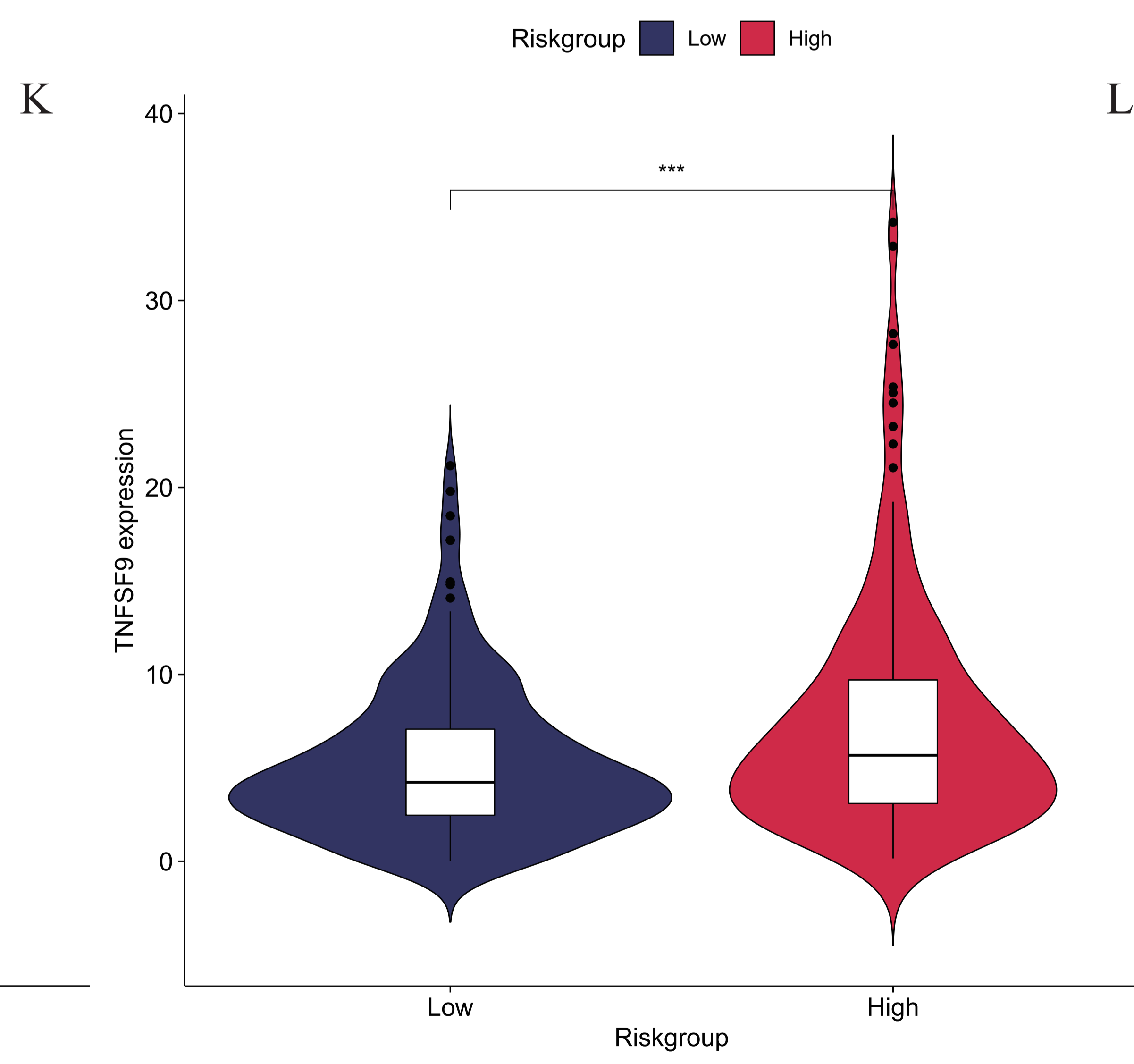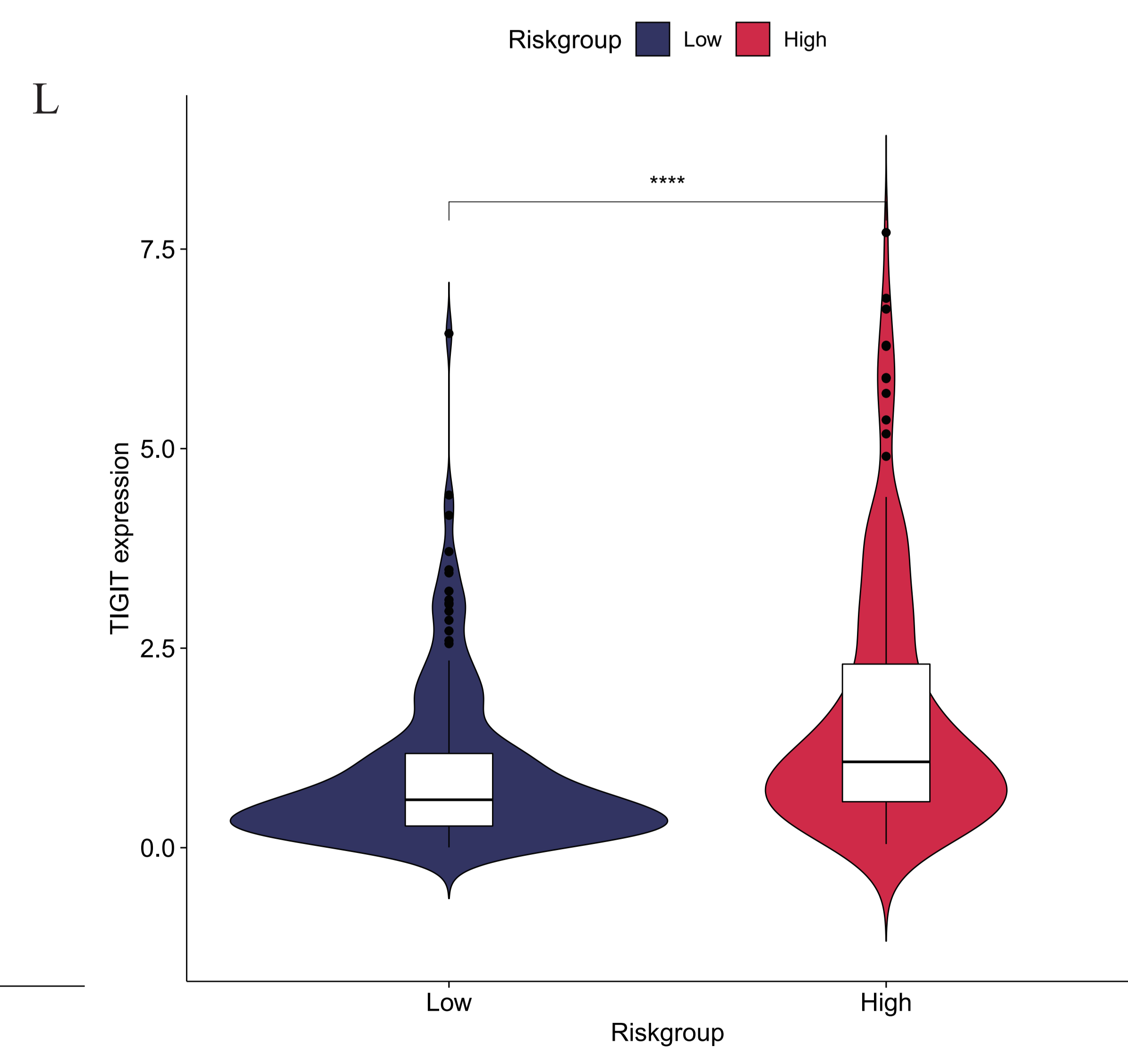

Supplement: Supplementary file 6 — Additional file 6: Figure S5. The relationship between prognostic signature and immune checkpoints. [file 40001_2023_1137_MOESM6_ESM.pdf]

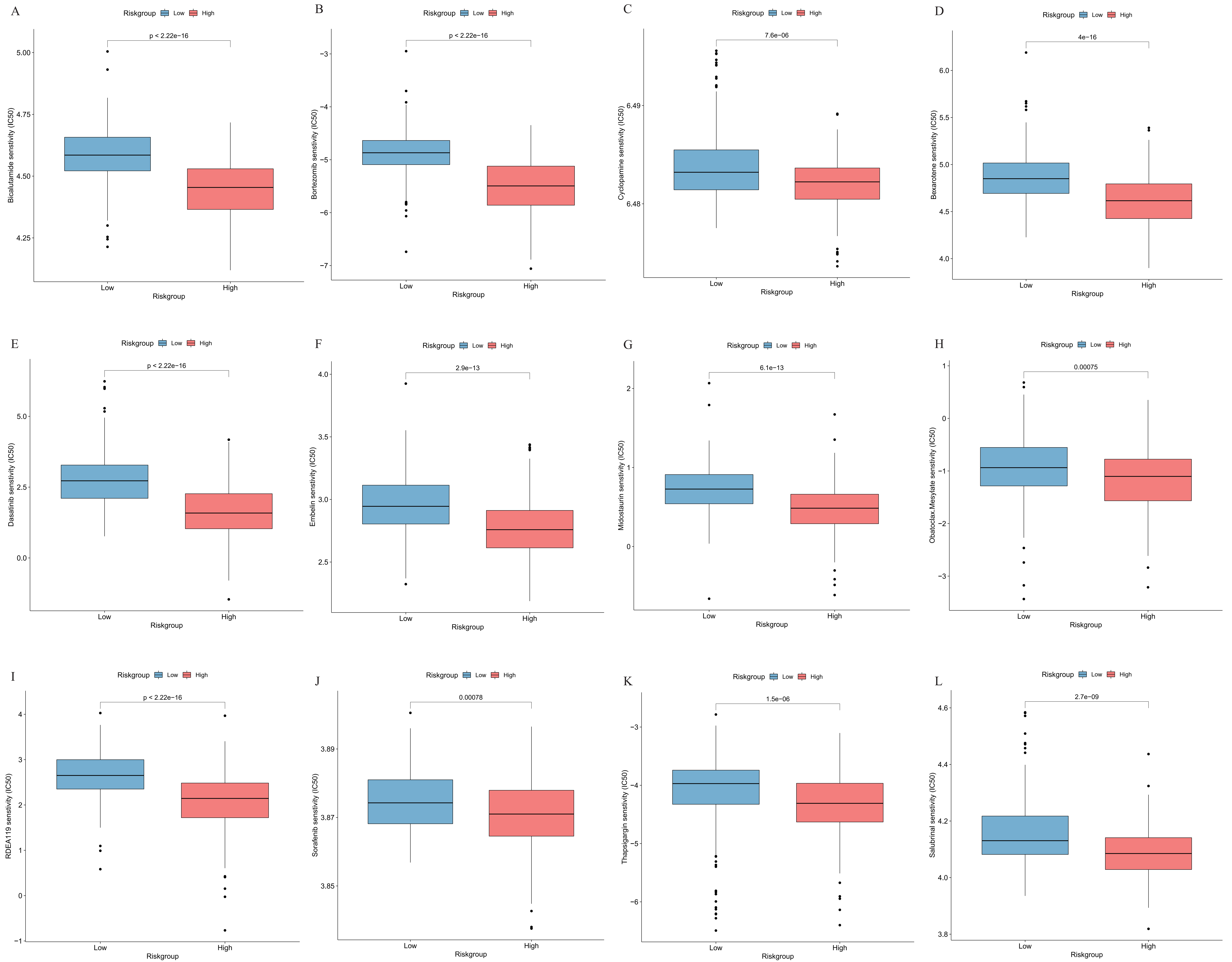

Supplement: Supplementary file 7 — Additional file 7: Figure S6. Drug sensitivity analysis. [file 40001_2023_1137_MOESM7_ESM.pdf]
